# Supplementary figures and images for: Insights into cargo sorting by SNX32 and its role in neurite outgrowth
Source: eLife. 2023 May 9;12:e84396. doi: 10.7554/eLife.84396 (PMC10219652; doi:10.7554/eLife.84396)

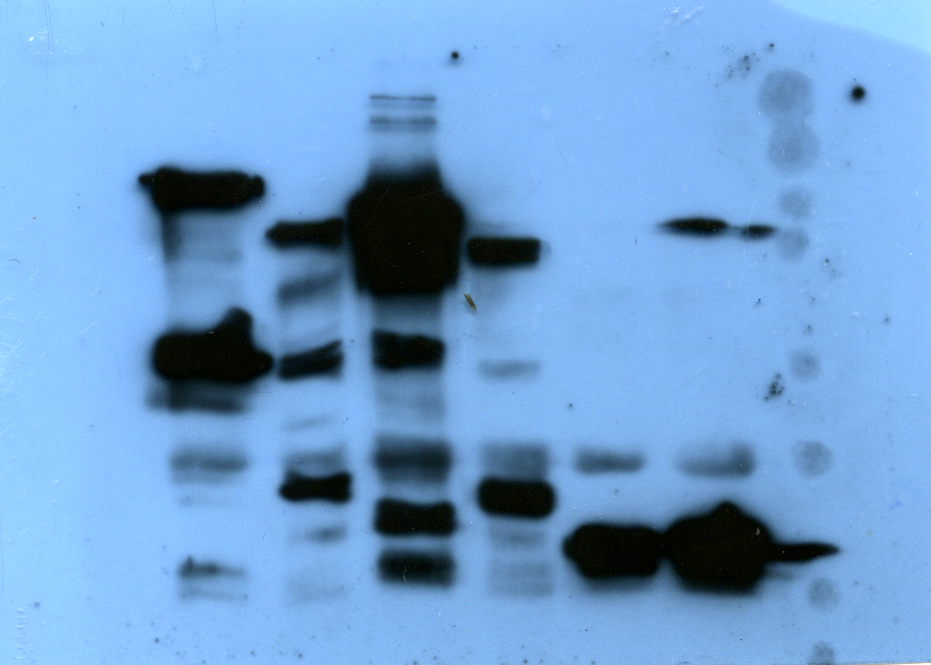

Supplement: Figure 1—source data 1. — Immunoblot source data of three biological replicates (values represent the ratio of HA to GFP band intensity). [file elife-84396-fig1-data1.zip › Figure1-source data 1/2.tif]

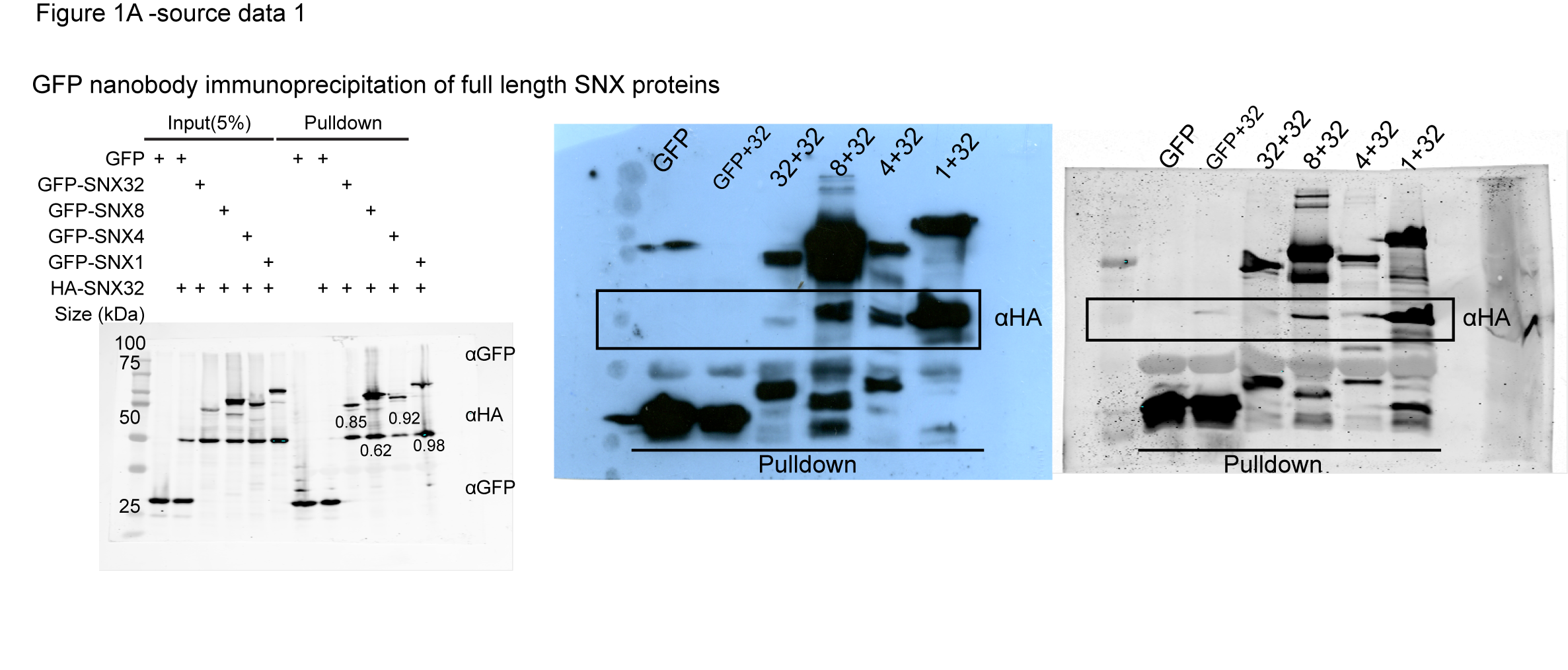

Supplement: Figure 1—source data 1. — Immunoblot source data of three biological replicates (values represent the ratio of HA to GFP band intensity). [file elife-84396-fig1-data1.zip › Figure1-source data 1/Figure1A-source data1.tif]

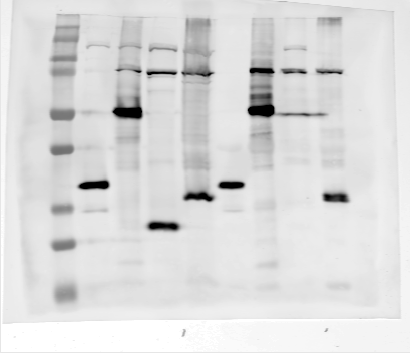

Supplement: Figure 1—source data 2. — Immunoblot source data of two biological replicates (values represent the ratio of HA to GFP band intensity). [file elife-84396-fig1-data2.zip › Figure1-source data 2/2.tif]

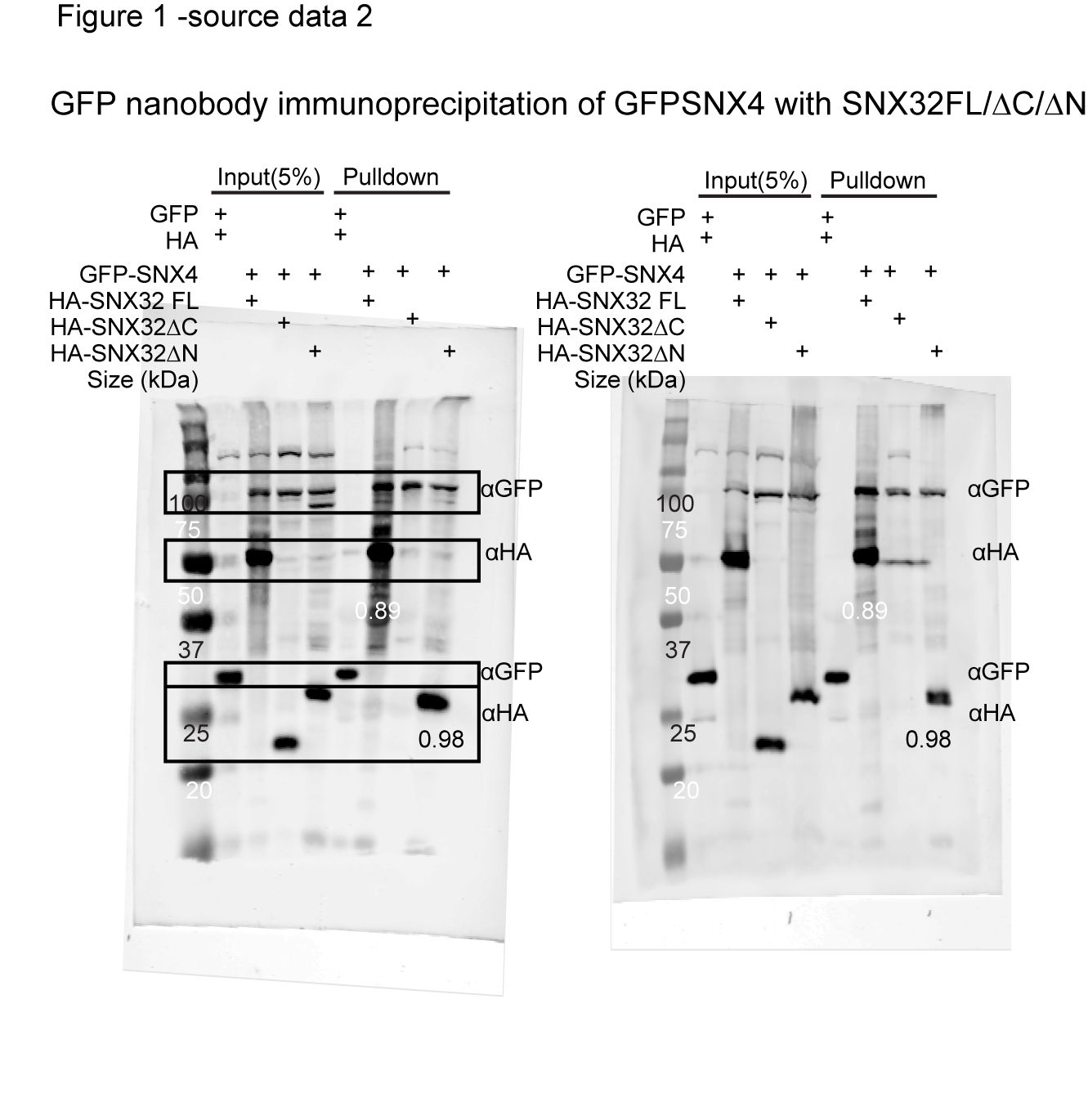

Supplement: Figure 1—source data 2. — Immunoblot source data of two biological replicates (values represent the ratio of HA to GFP band intensity). [file elife-84396-fig1-data2.zip › Figure1-source data 2/Figure 1 - source file 2.tif]

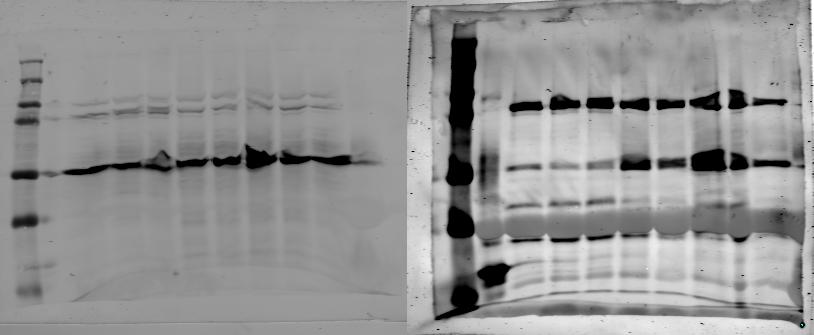

Supplement: Figure 1—source data 3. — Immunoblot source data of three biological replicates (values represent the ratio of HA to GFP band intensity). [file elife-84396-fig1-data3.zip › Figure1-source data 3/3.tif]

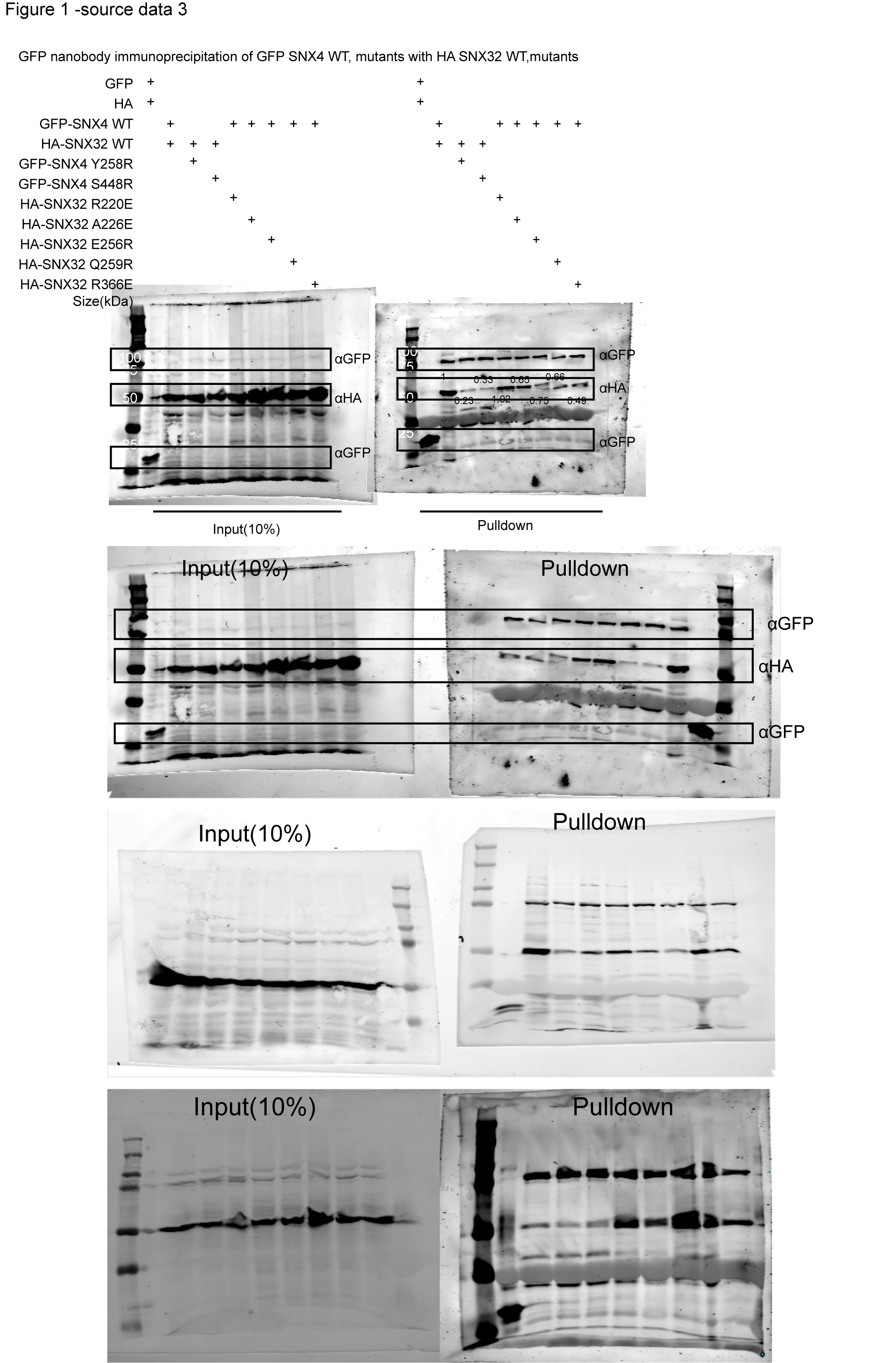

Supplement: Figure 1—source data 3. — Immunoblot source data of three biological replicates (values represent the ratio of HA to GFP band intensity). [file elife-84396-fig1-data3.zip › Figure1-source data 3/Figure1-source data3.tif]

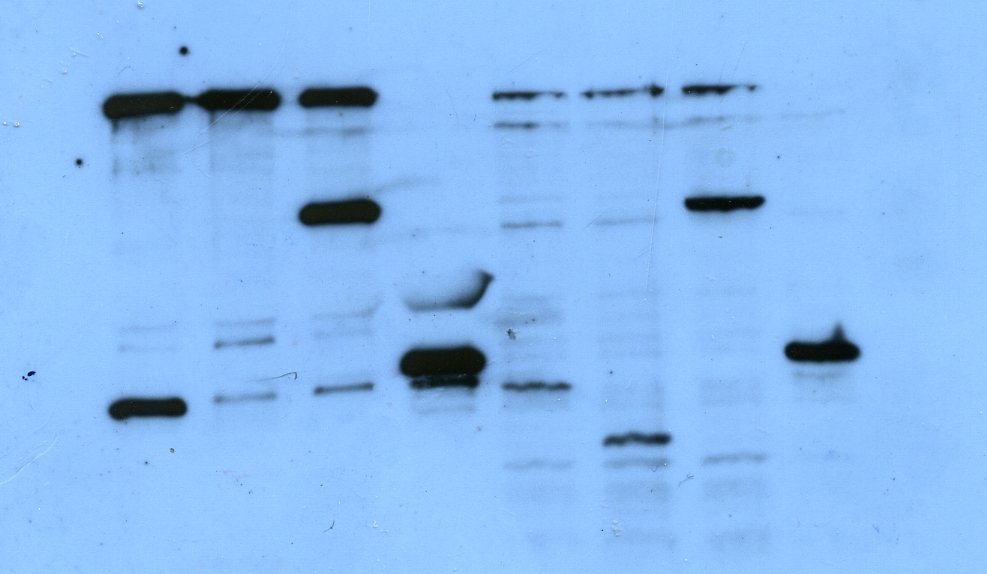

Supplement: Figure 1—figure supplement 1—source data 1. — GBP immunoprecipitation was carried out as described in the ‘Materials and methods’ section and immunoblotted using GFP and HA antibody. Immunoblot source data of two biological replicates. [file elife-84396-fig1-figsupp1-data1.zip › Figure 1- Figure Supplement 1-source data 1/1.jpg]

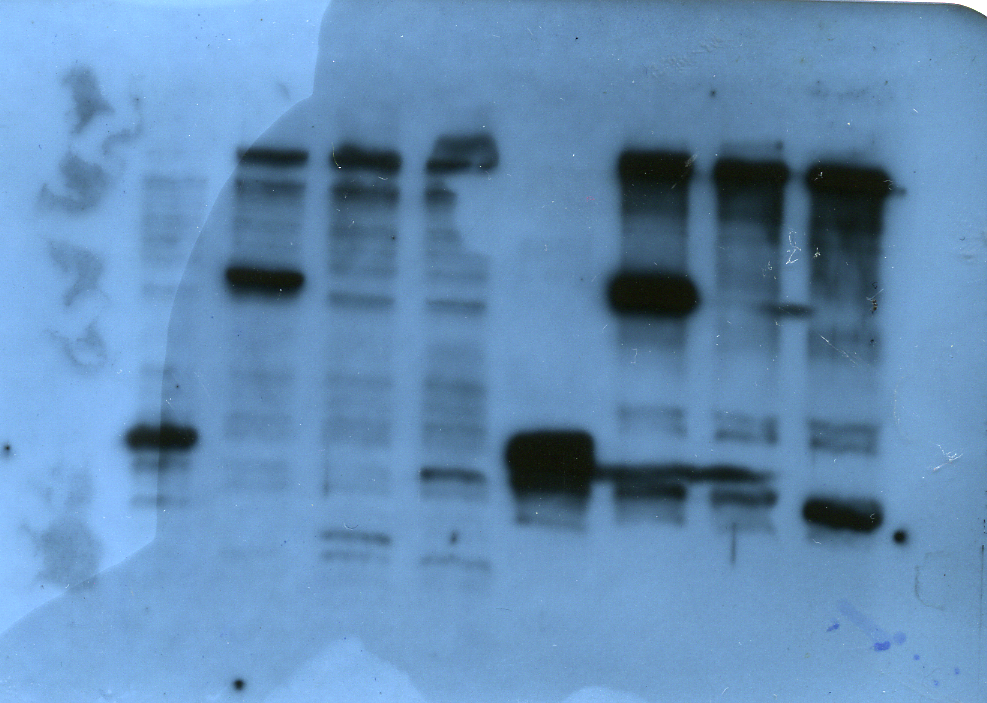

Supplement: Figure 1—figure supplement 1—source data 1. — GBP immunoprecipitation was carried out as described in the ‘Materials and methods’ section and immunoblotted using GFP and HA antibody. Immunoblot source data of two biological replicates. [file elife-84396-fig1-figsupp1-data1.zip › Figure 1- Figure Supplement 1-source data 1/2.tif]

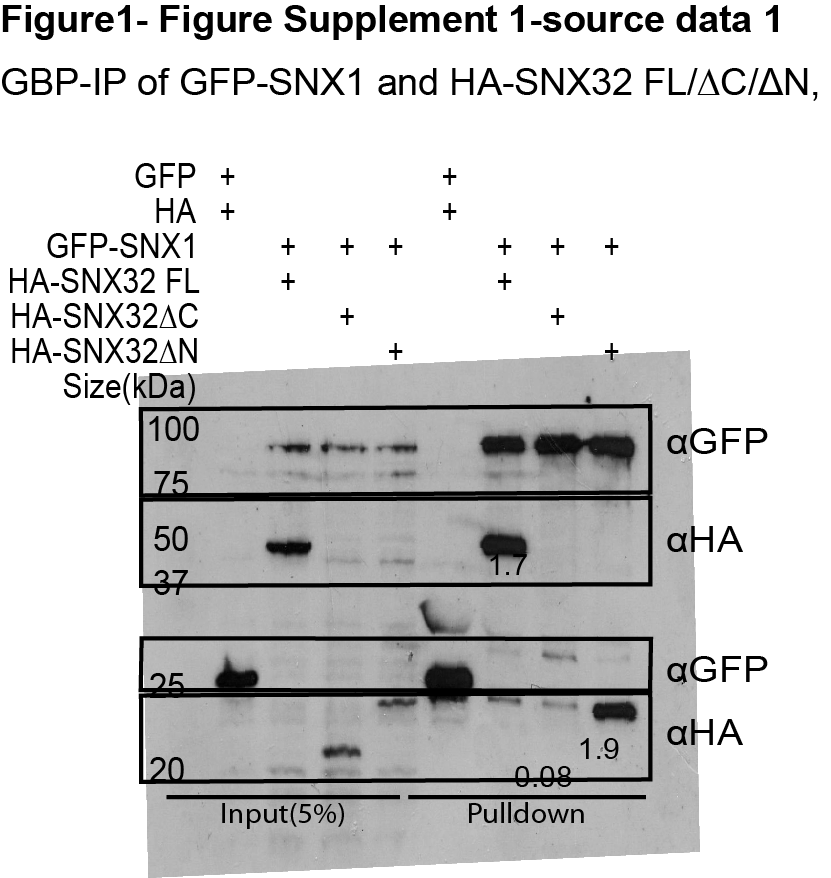

Supplement: Figure 1—figure supplement 1—source data 1. — GBP immunoprecipitation was carried out as described in the ‘Materials and methods’ section and immunoblotted using GFP and HA antibody. Immunoblot source data of two biological replicates. [file elife-84396-fig1-figsupp1-data1.zip › Figure 1- Figure Supplement 1-source data 1/Figure1-figure supplement 1-source data1.tif]

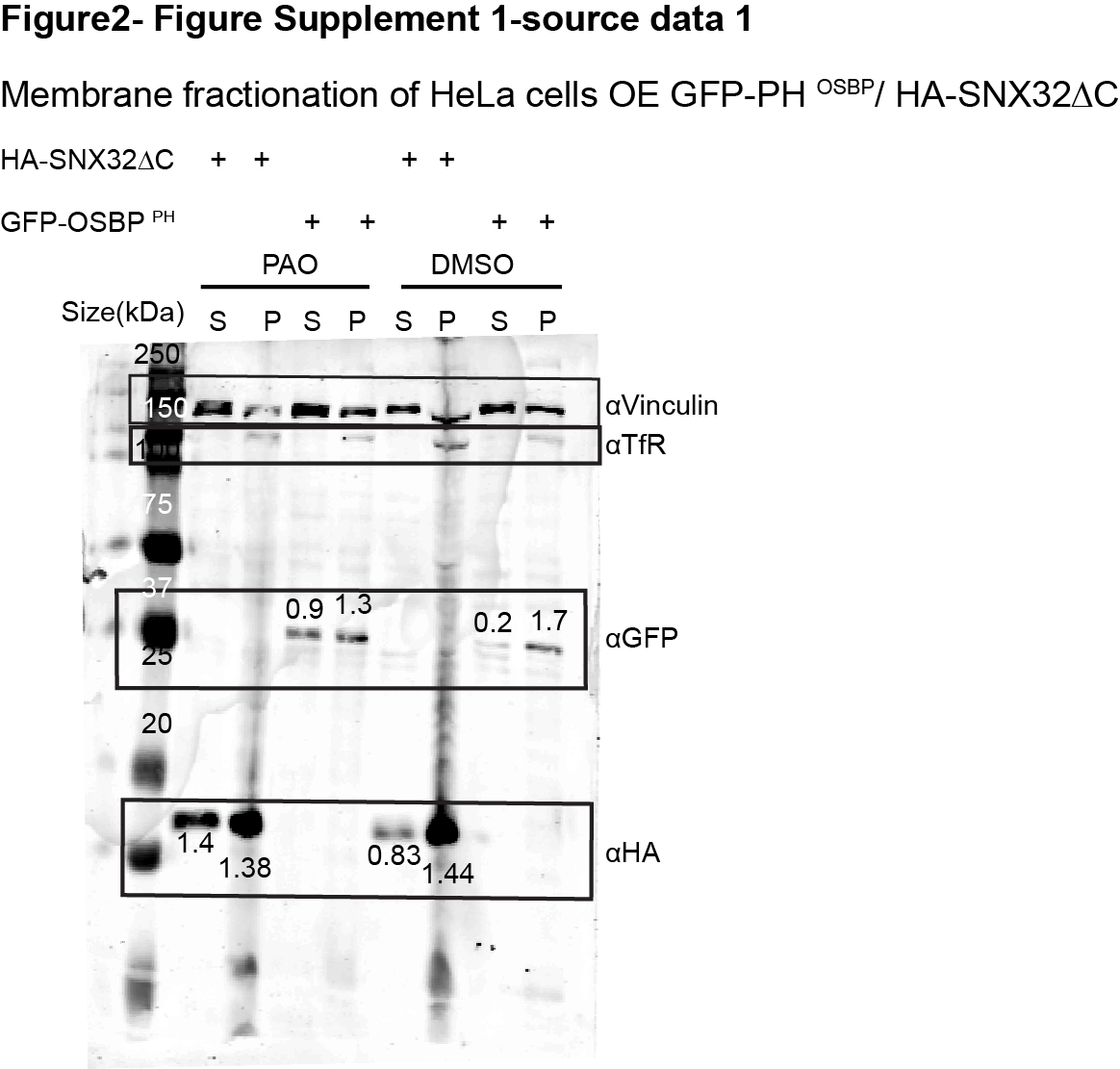

Supplement: Figure 2—figure supplement 1—source data 1. — Immunoblot source data (values represent the ratio of S to P fractions normalized to vinculin). [file elife-84396-fig2-figsupp1-data1.zip › Figure 2-figure supplement 1-source data1/Figure2-figure supplement 1-source data1.tif]

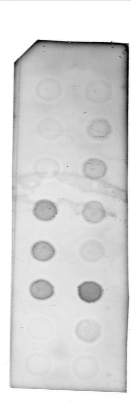

Supplement: Figure 2—figure supplement 2—source data 1. — Immunoblot source data of three biological replicates. [file elife-84396-fig2-figsupp2-data1.zip › Figure 2-Figure supplement 2- source data 1/1.tif]

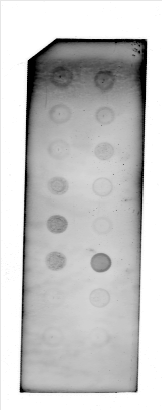

Supplement: Figure 2—figure supplement 2—source data 1. — Immunoblot source data of three biological replicates. [file elife-84396-fig2-figsupp2-data1.zip › Figure 2-Figure supplement 2- source data 1/2.tif]

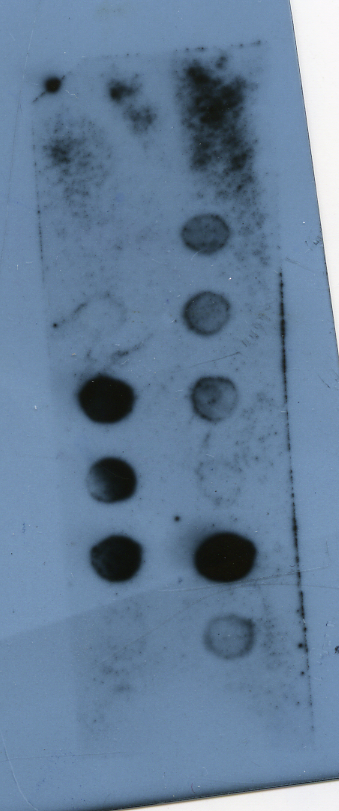

Supplement: Figure 2—figure supplement 2—source data 1. — Immunoblot source data of three biological replicates. [file elife-84396-fig2-figsupp2-data1.zip › Figure 2-Figure supplement 2- source data 1/3.tif]

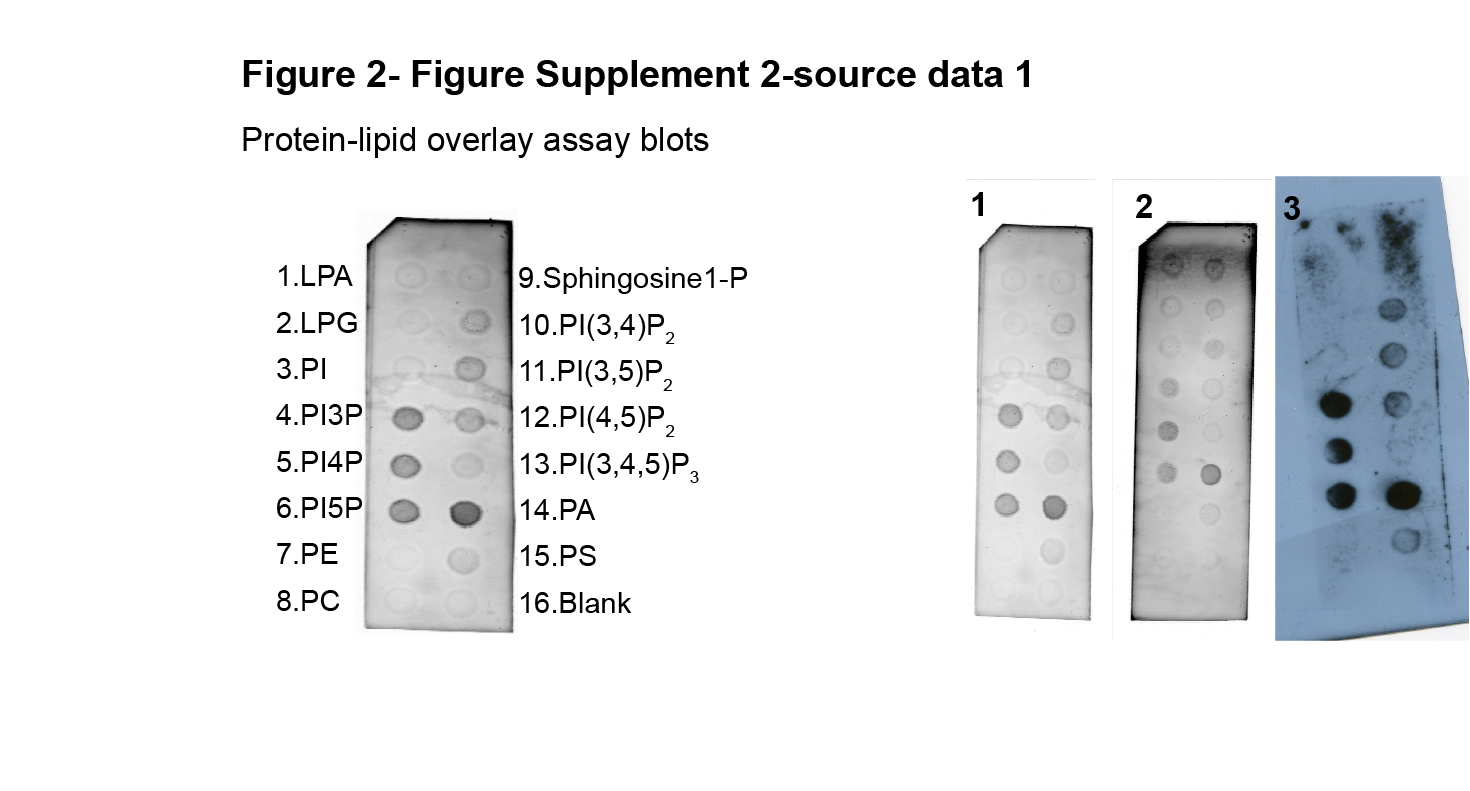

Supplement: Figure 2—figure supplement 2—source data 1. — Immunoblot source data of three biological replicates. [file elife-84396-fig2-figsupp2-data1.zip › Figure 2-Figure supplement 2- source data 1/Figure2-Figure supplement 2- source data 1.tif]

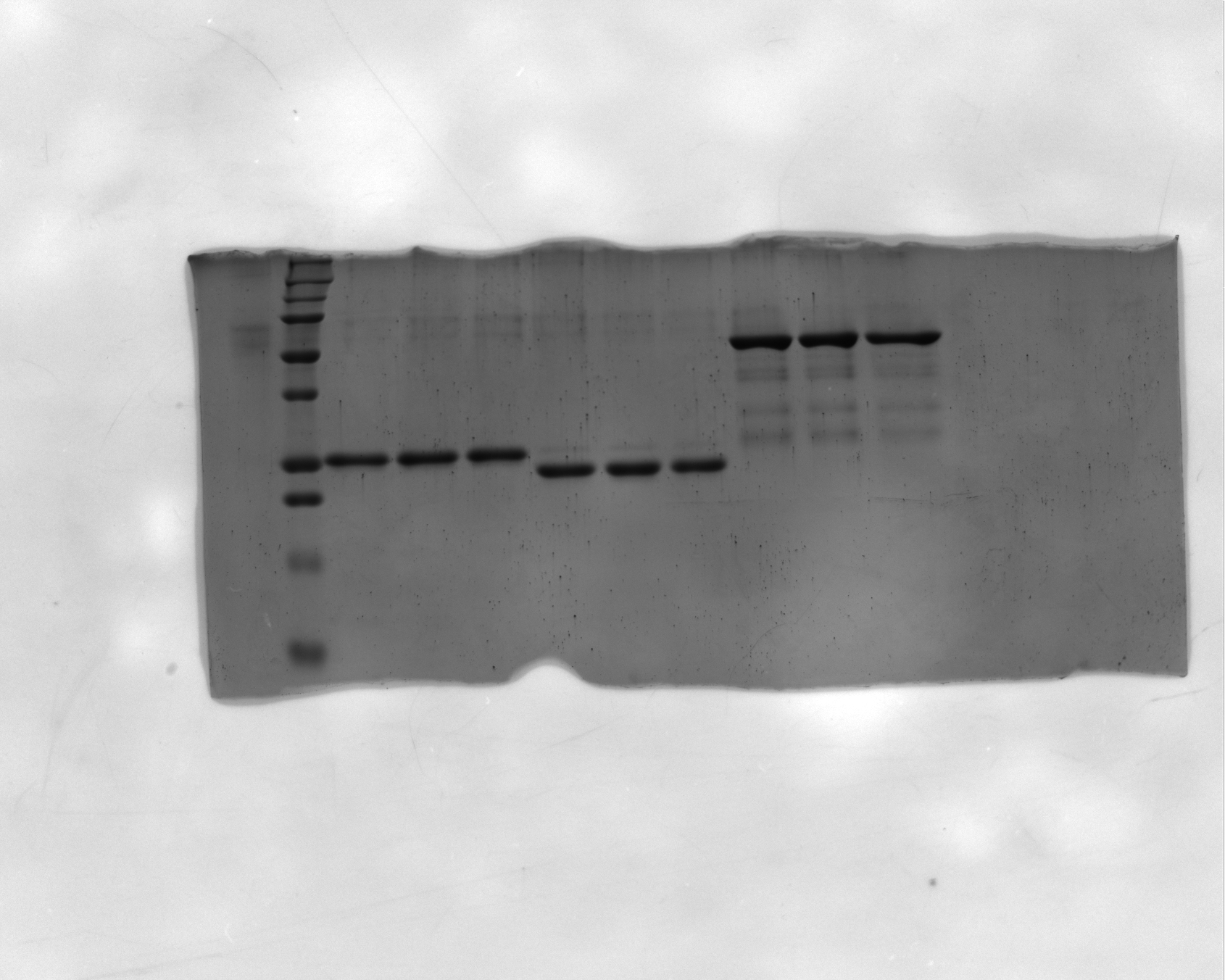

Supplement: Figure 2—figure supplement 2—source data 2. — Source data of three biological replicates. [file elife-84396-fig2-figsupp2-data2.zip › Figure 2-Figure supplement 2- source data 2/1/PROTEIN_GEL_02092023_114723_(Protein Gel)_raw.tif]

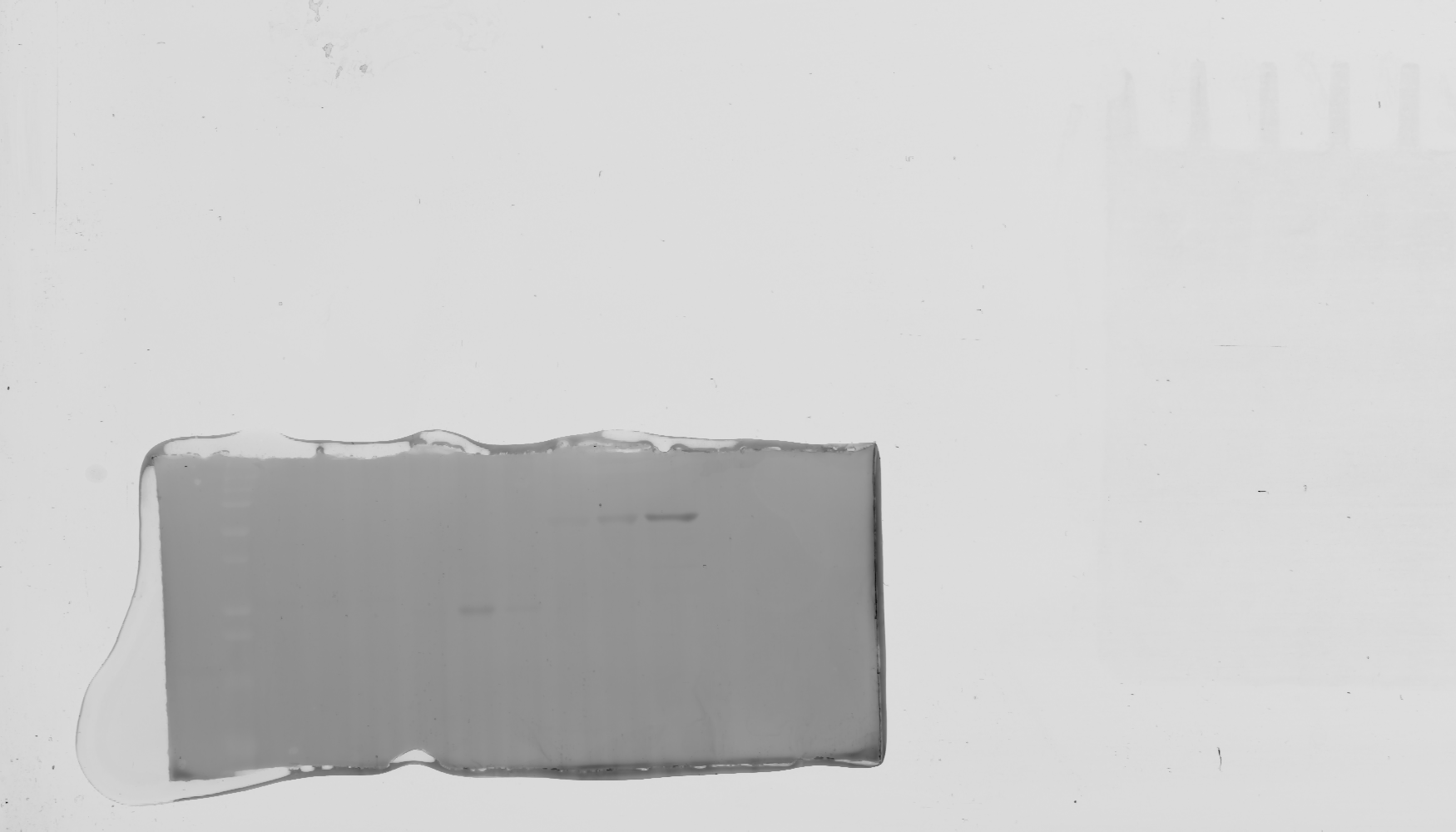

Supplement: Figure 2—figure supplement 2—source data 2. — Source data of three biological replicates. [file elife-84396-fig2-figsupp2-data2.zip › Figure 2-Figure supplement 2- source data 2/1/SNX32_PliMAP1_fluor2-[Cy2].tif]

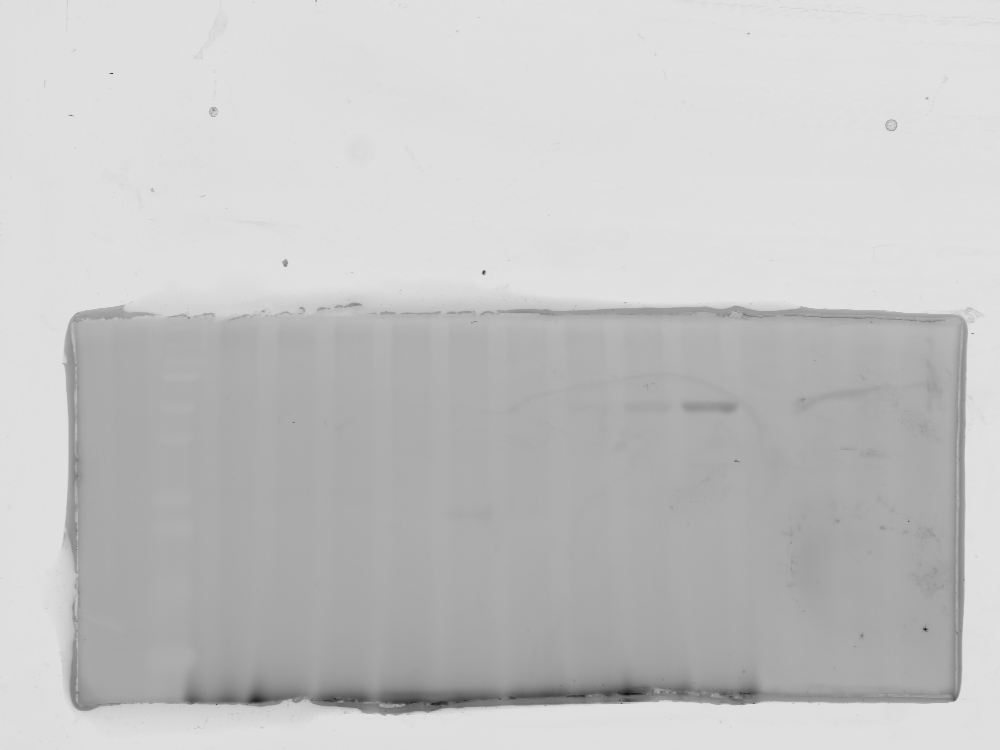

Supplement: Figure 2—figure supplement 2—source data 2. — Source data of three biological replicates. [file elife-84396-fig2-figsupp2-data2.zip › Figure 2-Figure supplement 2- source data 2/2/20230209-SNX32_PliMAP_2-[Cy2].tif]

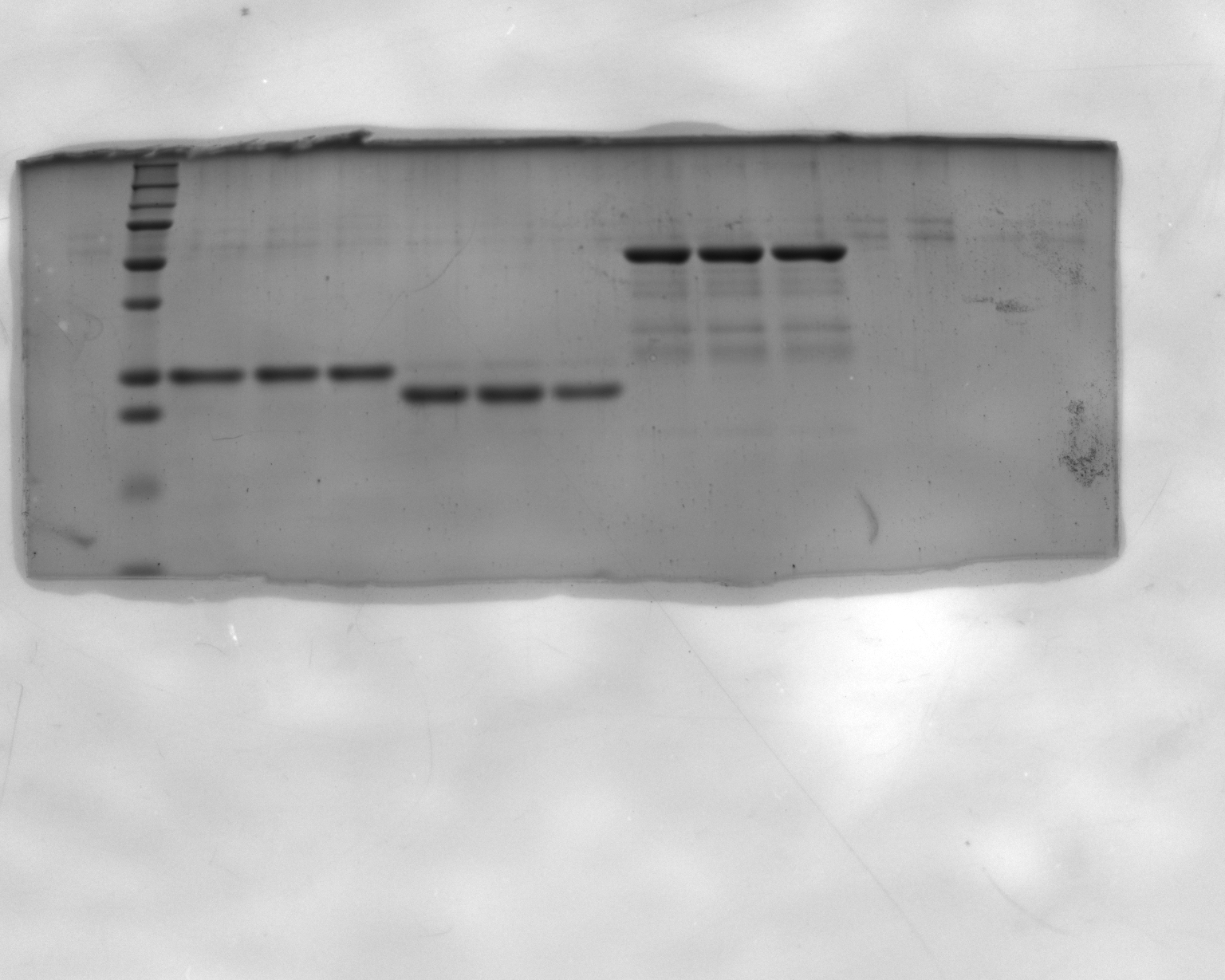

Supplement: Figure 2—figure supplement 2—source data 2. — Source data of three biological replicates. [file elife-84396-fig2-figsupp2-data2.zip › Figure 2-Figure supplement 2- source data 2/2/PROTEIN_GEL_02112023_140437_(Protein Gel)_raw.tif]

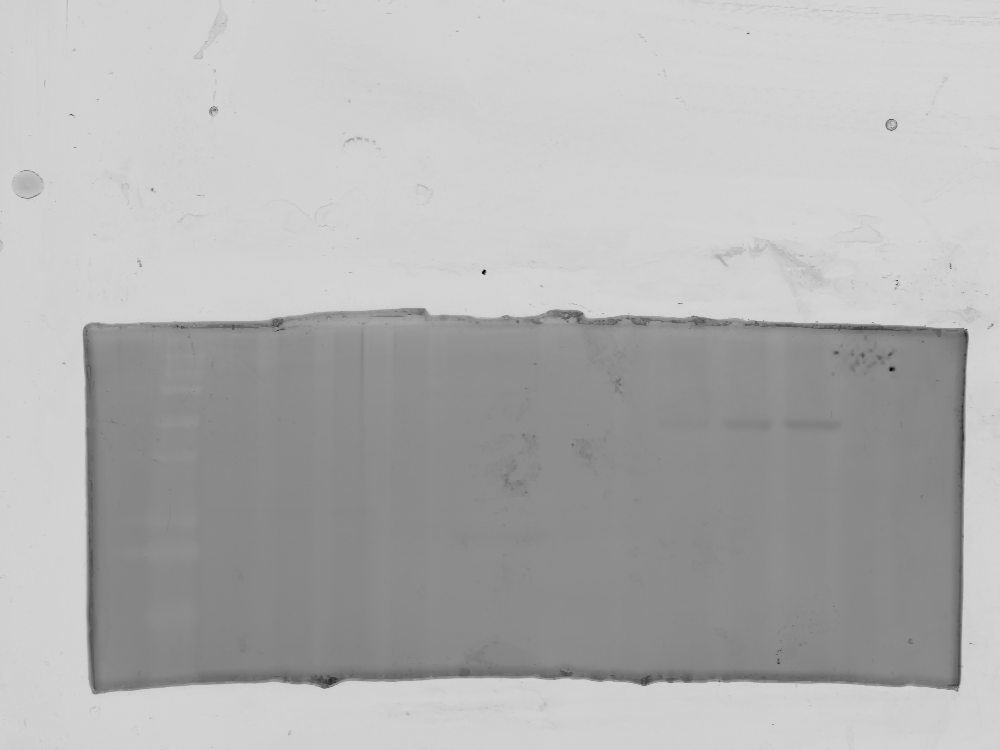

Supplement: Figure 2—figure supplement 2—source data 2. — Source data of three biological replicates. [file elife-84396-fig2-figsupp2-data2.zip › Figure 2-Figure supplement 2- source data 2/3/20230209-SNX32_PliMAP_3_invert-[Cy2].tif]

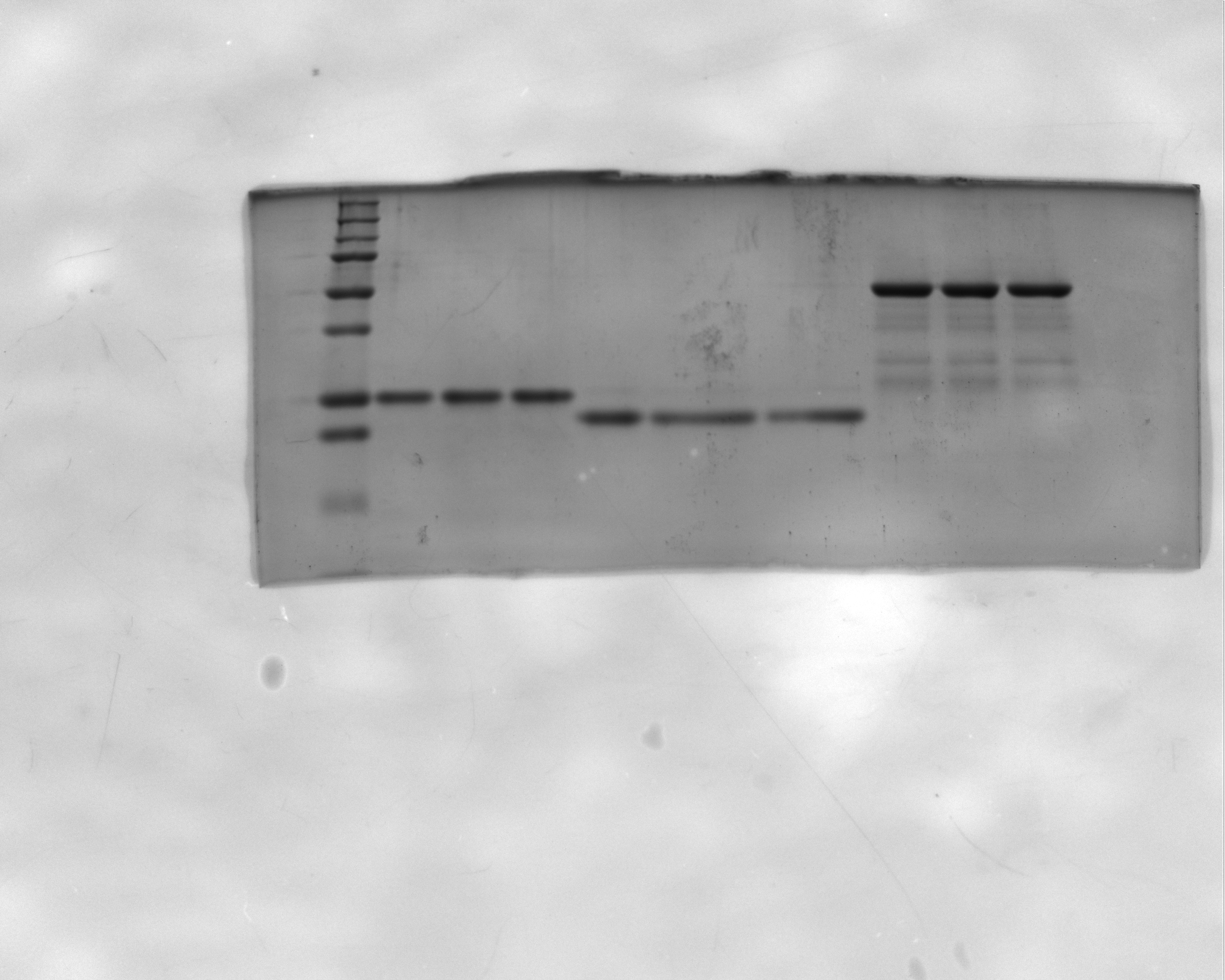

Supplement: Figure 2—figure supplement 2—source data 2. — Source data of three biological replicates. [file elife-84396-fig2-figsupp2-data2.zip › Figure 2-Figure supplement 2- source data 2/3/PROTEIN_GEL_02112023_140246_(Protein Gel)_raw.tif]

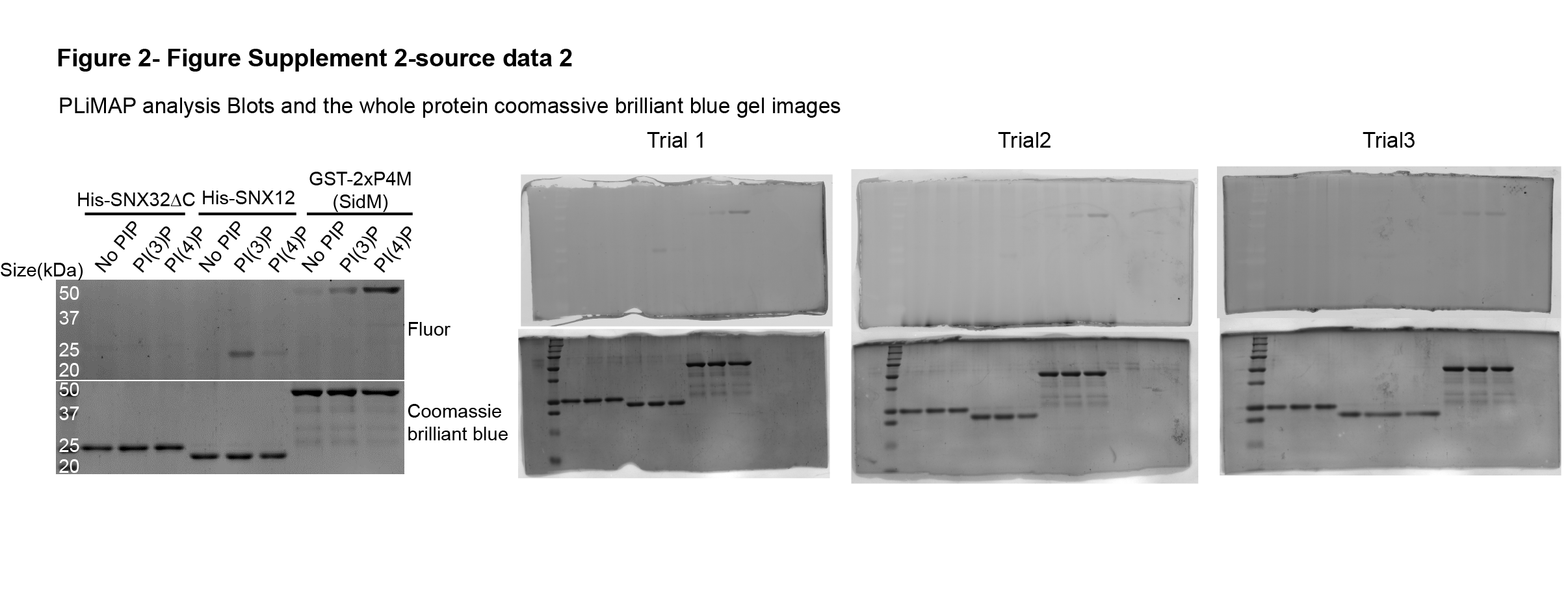

Supplement: Figure 2—figure supplement 2—source data 2. — Source data of three biological replicates. [file elife-84396-fig2-figsupp2-data2.zip › Figure 2-Figure supplement 2- source data 2/Figure 2- Figure Supplement 2-source data 2.tif]

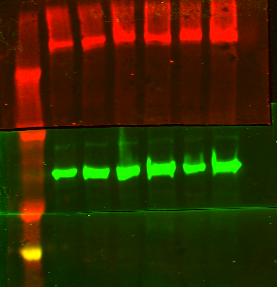

Supplement: Figure 3—figure supplement 1—source data 1. — Immunoblotting was done using CIMPR or vinculin antibody. Immunoblot source data of three biological replicates. [file elife-84396-fig3-figsupp1-data1.zip › Figure 3- figure supplement 1- source data 1/1.tif]

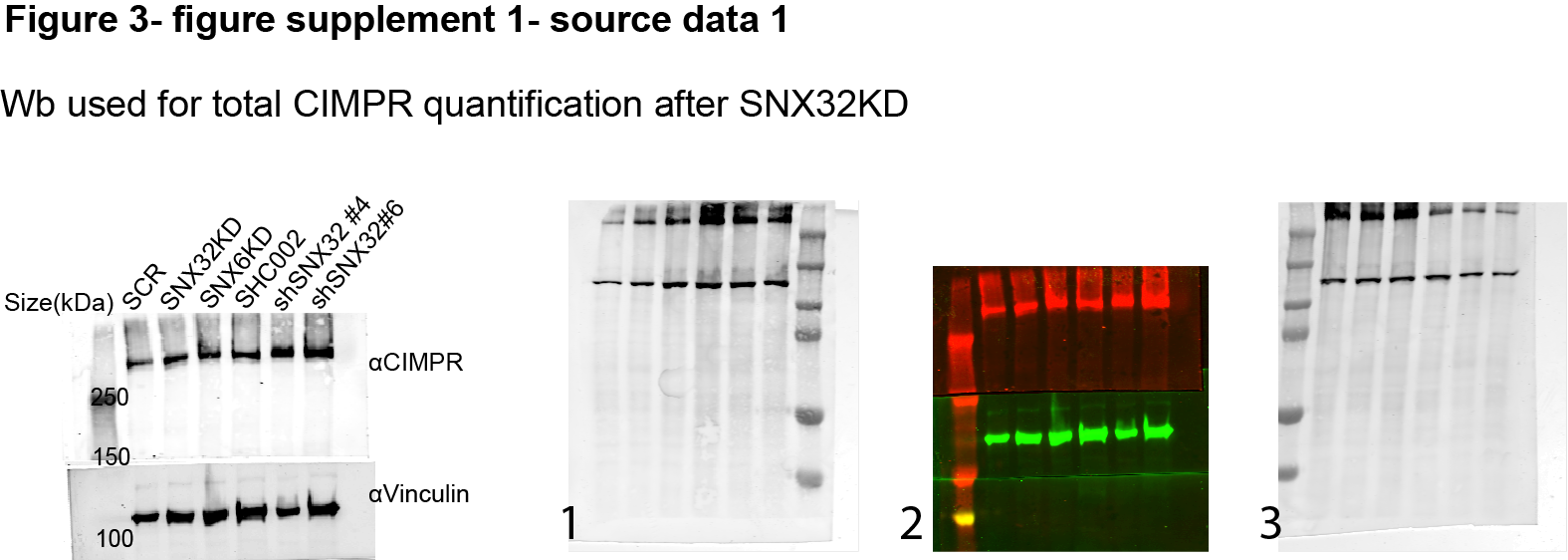

Supplement: Figure 3—figure supplement 1—source data 1. — Immunoblotting was done using CIMPR or vinculin antibody. Immunoblot source data of three biological replicates. [file elife-84396-fig3-figsupp1-data1.zip › Figure 3- figure supplement 1- source data 1/Figure 3- figure supplement 1- source data 1.tif]

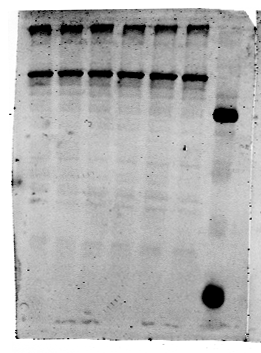

Supplement: Figure 4—figure supplement 2—source data 1. — Immunoblot source data of three biological replicates. [file elife-84396-fig4-figsupp2-data1.zip › Figure 4- figure supplement 2- source data 1/1.tif]

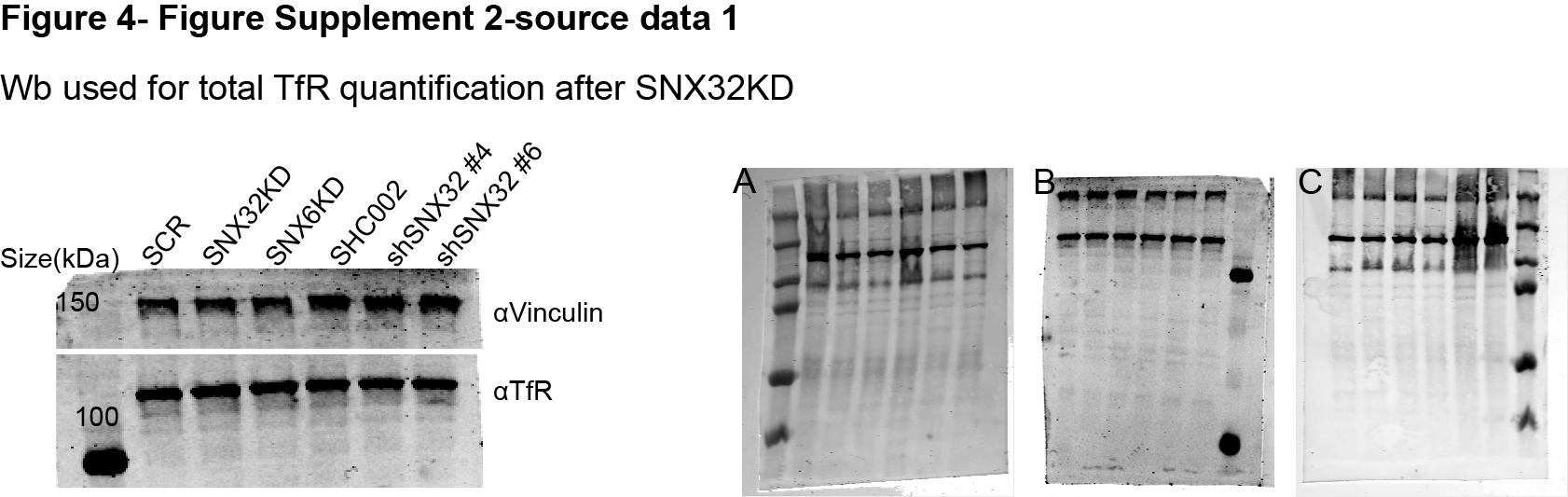

Supplement: Figure 4—figure supplement 2—source data 1. — Immunoblot source data of three biological replicates. [file elife-84396-fig4-figsupp2-data1.zip › Figure 4- figure supplement 2- source data 1/Figure 4- figure supplement 2- source data 1.tif]

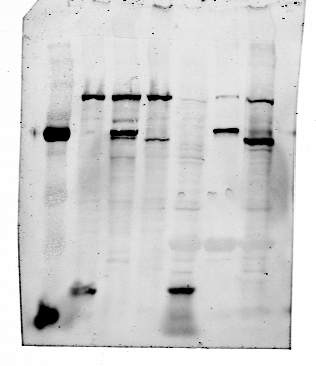

Supplement: Figure 5—source data 1. — GBP immunoprecipitation was carried out as described in the ‘Materials and methods’ section and immunoblotted using GFP and TfR antibody. Immunoblot source data (values represent the ratio of TfR to GFP band intensity). [file elife-84396-fig5-data1.zip › Figure5- source data 1/1.tif]

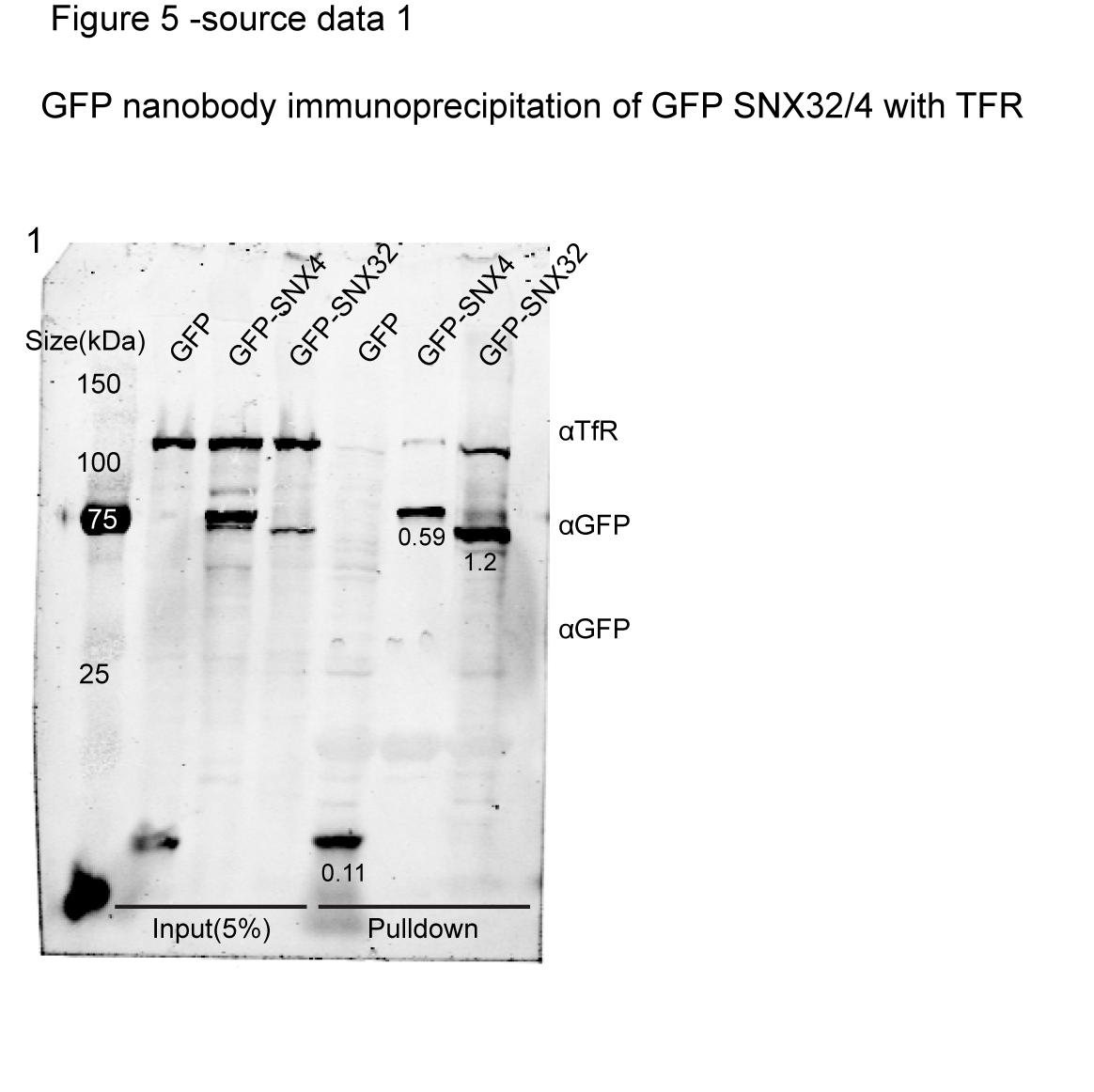

Supplement: Figure 5—source data 1. — GBP immunoprecipitation was carried out as described in the ‘Materials and methods’ section and immunoblotted using GFP and TfR antibody. Immunoblot source data (values represent the ratio of TfR to GFP band intensity). [file elife-84396-fig5-data1.zip › Figure5- source data 1/Figure5- source data 1.tif]

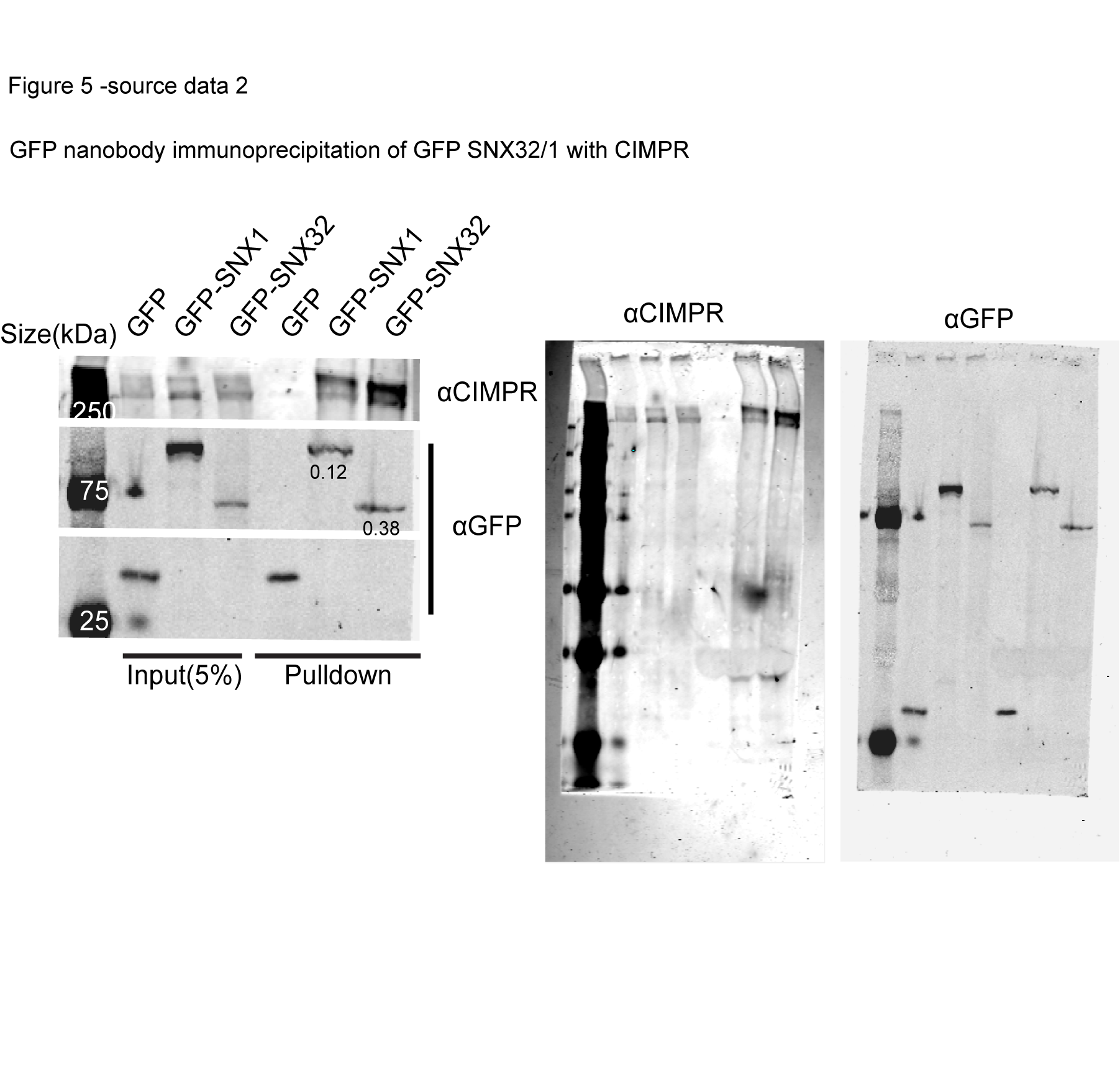

Supplement: Figure 5—source data 2. — GBP immunoprecipitation was carried out as described in the ‘Materials and methods’ section and immunoblotted using GFP and TfR antibody. Immunoblot source data (values represent the ratio of CIMPR to GFP band intensity). [file elife-84396-fig5-data2.zip › Figure5-source data 2/Figure5-source data 2.tif]

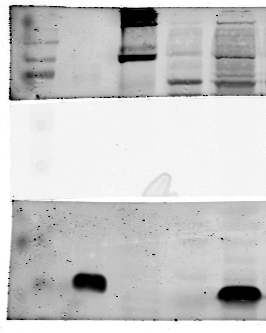

Supplement: Figure 5—source data 3. — His pulldown was carried out as described in the ‘Materials and methods’ section and immunoblotted using His and TfR antibody. Immunoblot source data of two biological replicates (values represent the ratio of His to TfR band intensity). [file elife-84396-fig5-data3.zip › Figure5-source data 3/1.tif]

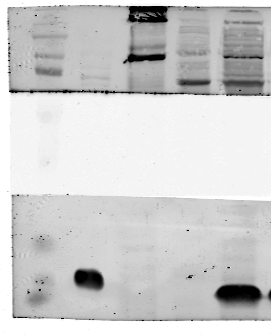

Supplement: Figure 5—source data 3. — His pulldown was carried out as described in the ‘Materials and methods’ section and immunoblotted using His and TfR antibody. Immunoblot source data of two biological replicates (values represent the ratio of His to TfR band intensity). [file elife-84396-fig5-data3.zip › Figure5-source data 3/2.tif]

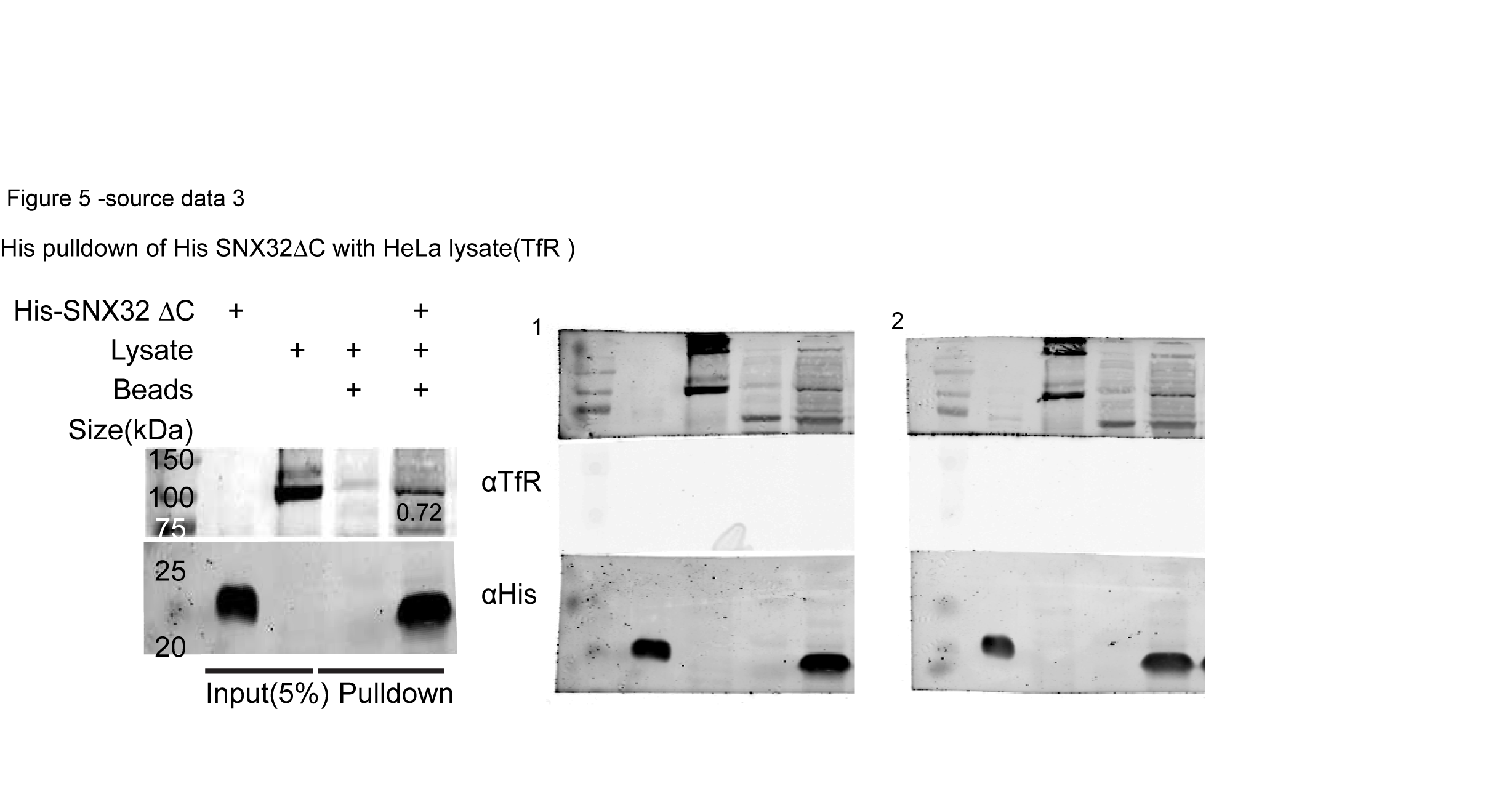

Supplement: Figure 5—source data 3. — His pulldown was carried out as described in the ‘Materials and methods’ section and immunoblotted using His and TfR antibody. Immunoblot source data of two biological replicates (values represent the ratio of His to TfR band intensity). [file elife-84396-fig5-data3.zip › Figure5-source data 3/Figure5-souce data3.tif]

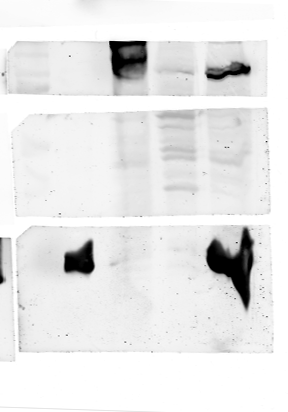

Supplement: Figure 5—source data 4. — His pulldown was carried out as described in the ‘Materials and methods’ section and immunoblotted using His and TfR antibody. Immunoblot source data. [file elife-84396-fig5-data4.zip › Figure5-source data 4/1.tif]

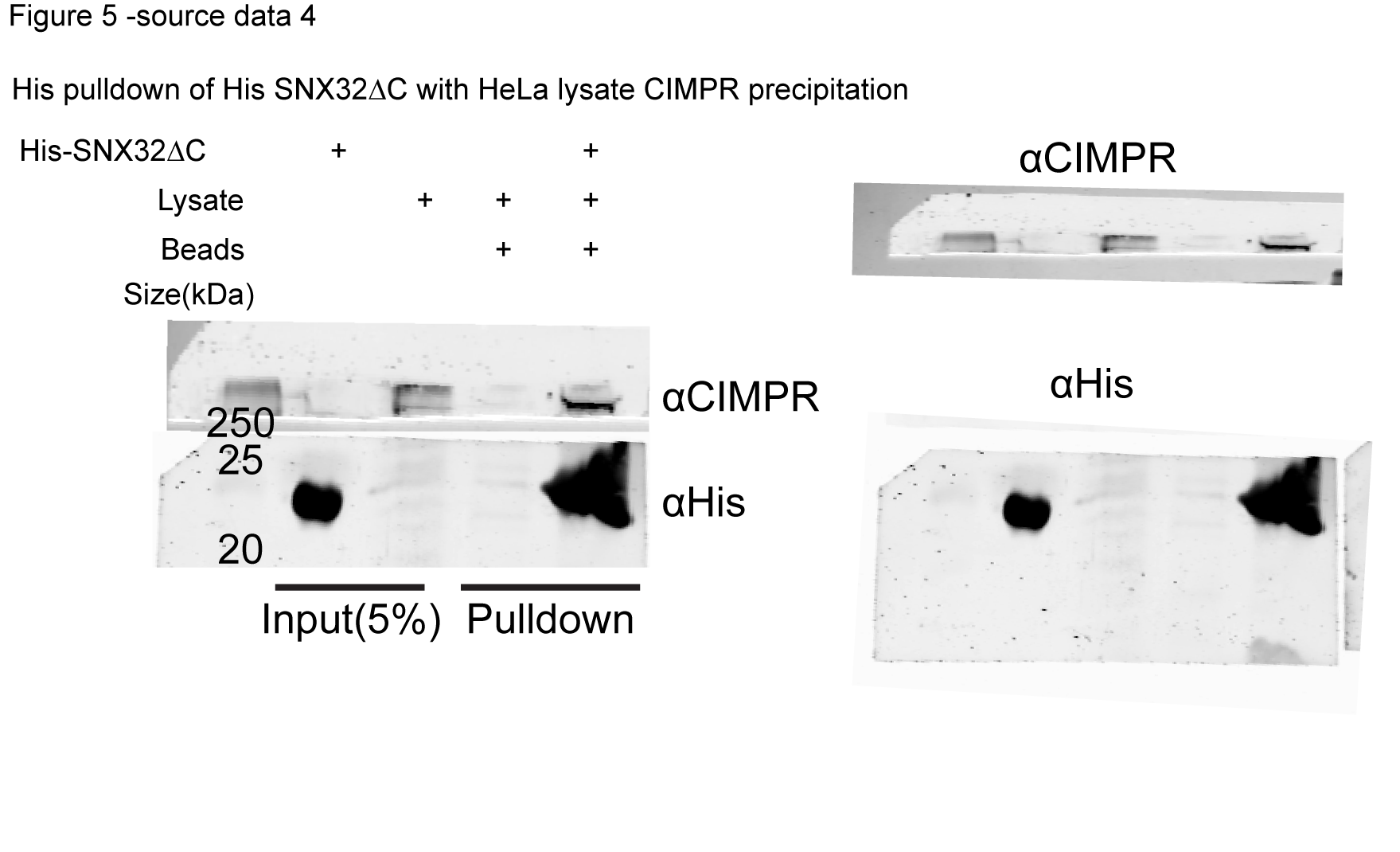

Supplement: Figure 5—source data 4. — His pulldown was carried out as described in the ‘Materials and methods’ section and immunoblotted using His and TfR antibody. Immunoblot source data. [file elife-84396-fig5-data4.zip › Figure5-source data 4/Figure5-souce data4.tif]

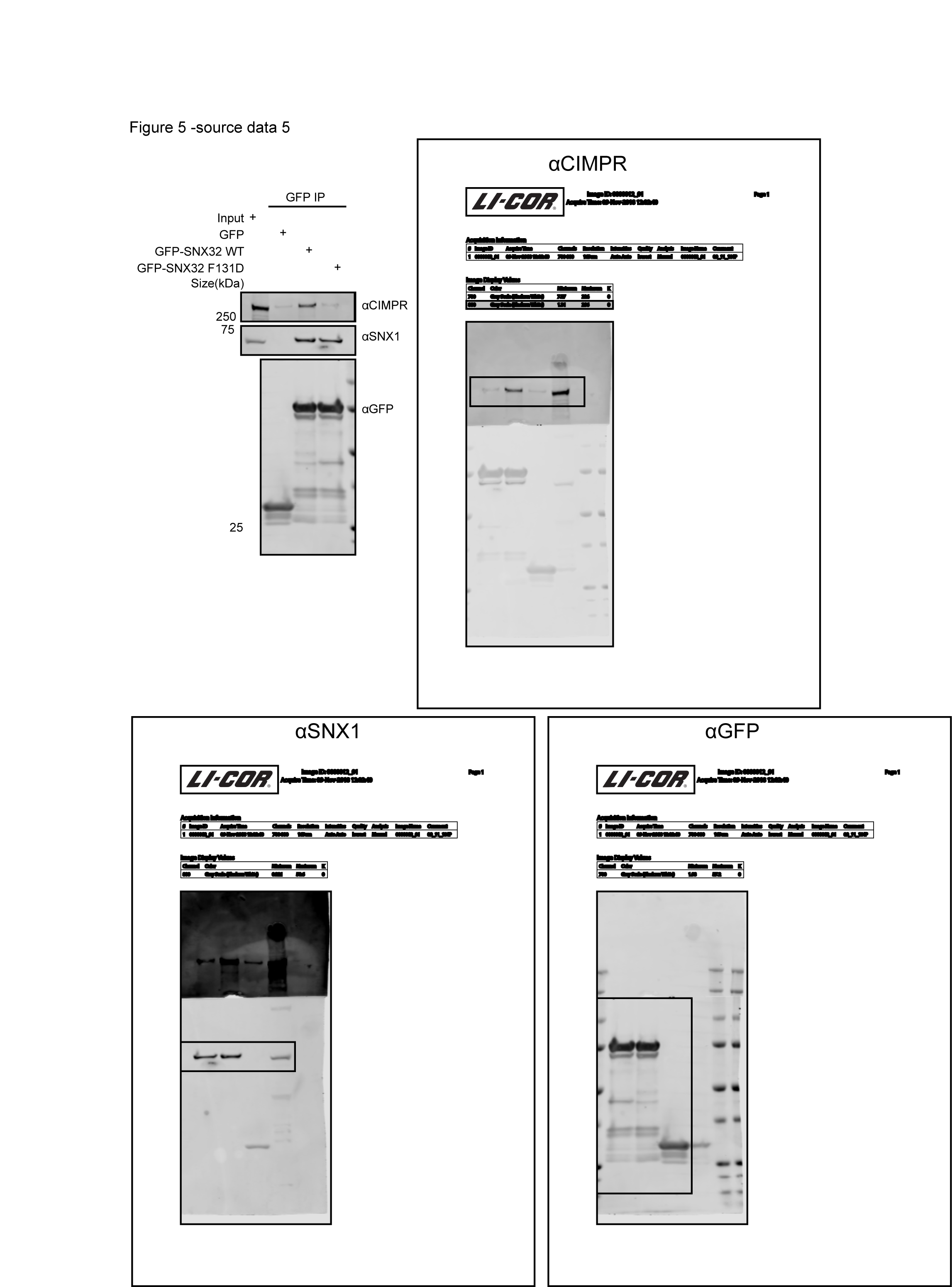

Supplement: Figure 5—source data 5. — The elute was resolved in SDS-PAGE and immunoblotted using GFP, SNX1, and CIMPR antibody. Immunoblot source data. [file elife-84396-fig5-data5.zip › Figure5-source data 5/Figure5-source data 5.tif]

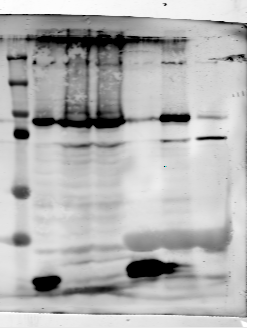

Supplement: Figure 5—source data 6. — GBP immunoprecipitation was carried out as described in the ‘Materials and methods’ section and immunoblotted using GFP and TfR antibody. Immunoblot source data of three biological replicates (values represent the ratio of TfR to GFP band intensity). [file elife-84396-fig5-data6.zip › Figure5-source data 6/2.tif]

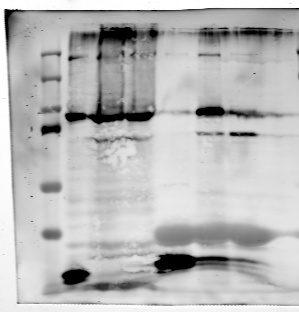

Supplement: Figure 5—source data 6. — GBP immunoprecipitation was carried out as described in the ‘Materials and methods’ section and immunoblotted using GFP and TfR antibody. Immunoblot source data of three biological replicates (values represent the ratio of TfR to GFP band intensity). [file elife-84396-fig5-data6.zip › Figure5-source data 6/3.tif]

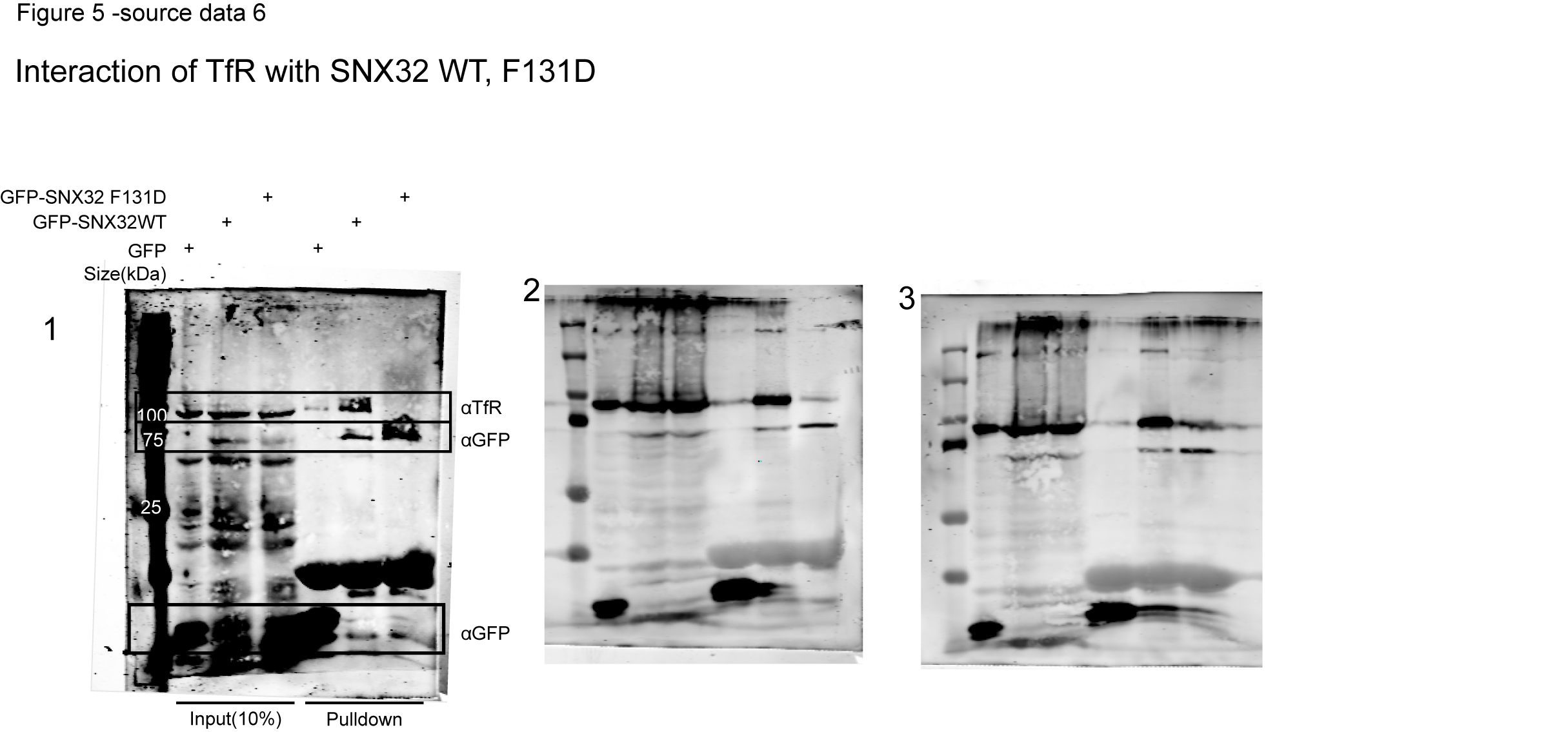

Supplement: Figure 5—source data 6. — GBP immunoprecipitation was carried out as described in the ‘Materials and methods’ section and immunoblotted using GFP and TfR antibody. Immunoblot source data of three biological replicates (values represent the ratio of TfR to GFP band intensity). [file elife-84396-fig5-data6.zip › Figure5-source data 6/Figure5-source data 6.tif]

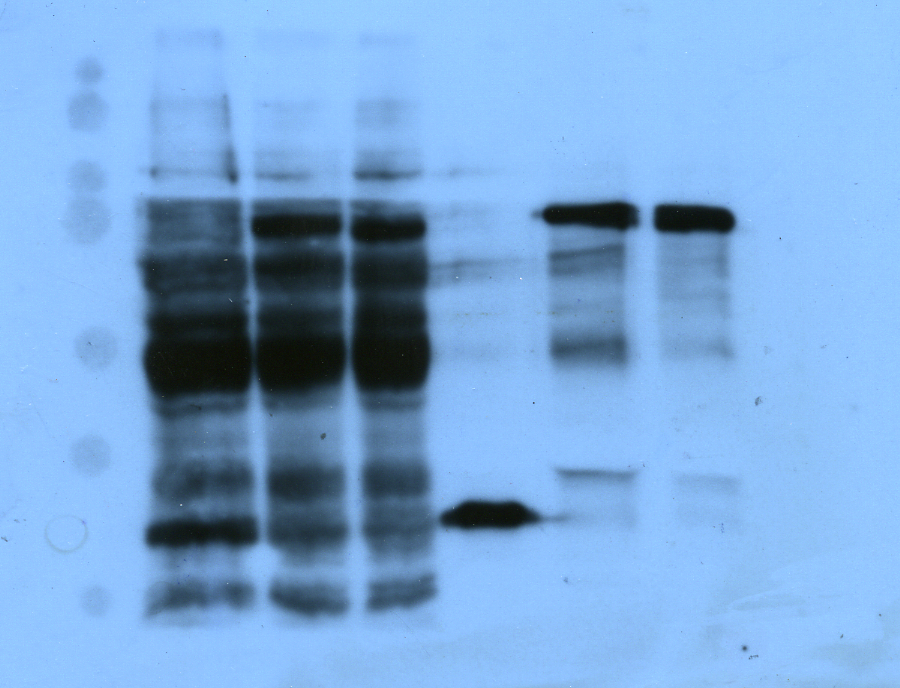

Supplement: Figure 6—source data 1. — mCherry nanobody-mediated pulldown was carried out as described in the ‘Materials and methods’ section and immunoblotted using mCherry and BSG antibody. Immunoblot source data of two biological replicates (values represent the ratio of BSG to mCherry band intensity). [file elife-84396-fig6-data1.zip › Figure6-source data 1/1.tif]

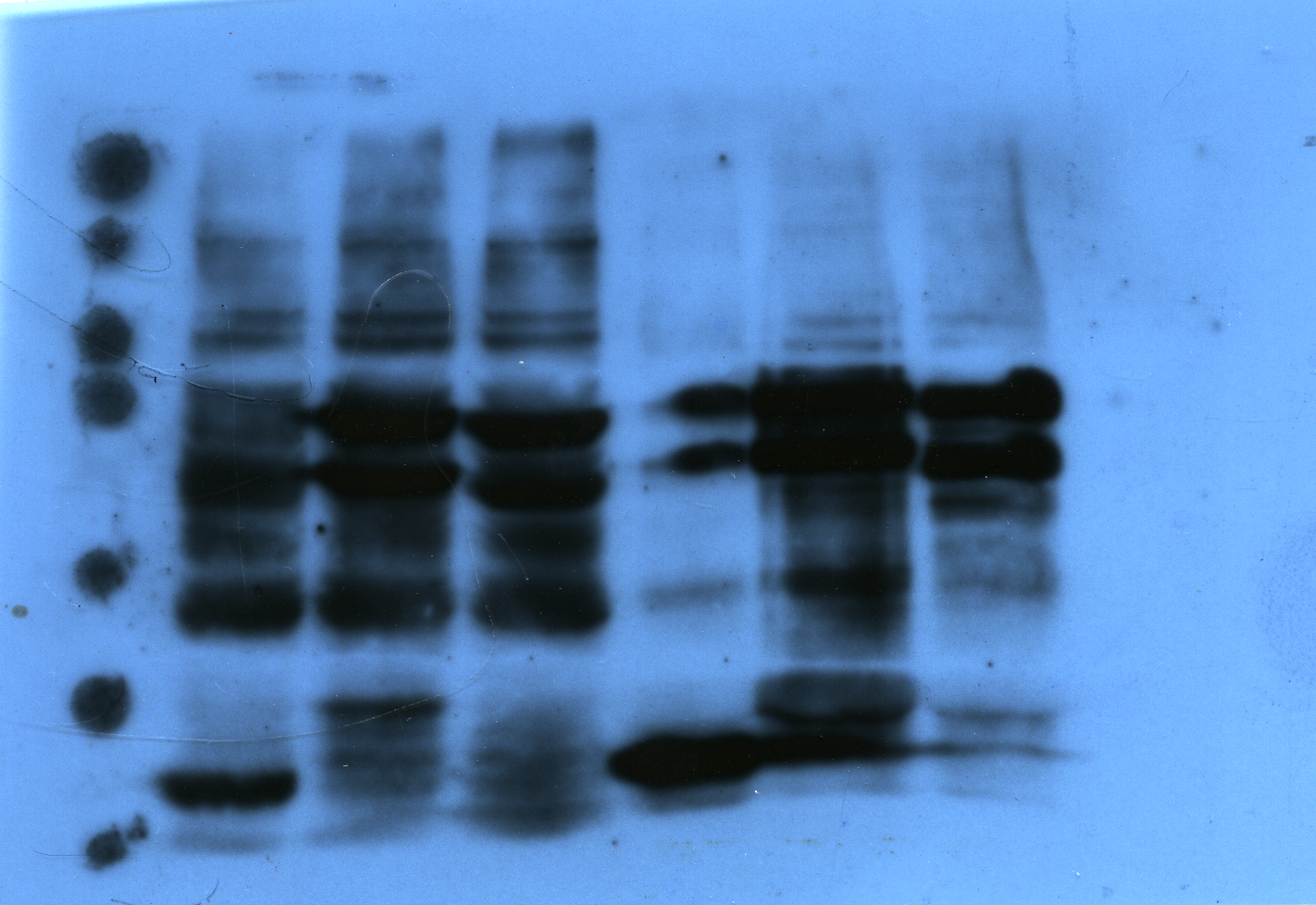

Supplement: Figure 6—source data 1. — mCherry nanobody-mediated pulldown was carried out as described in the ‘Materials and methods’ section and immunoblotted using mCherry and BSG antibody. Immunoblot source data of two biological replicates (values represent the ratio of BSG to mCherry band intensity). [file elife-84396-fig6-data1.zip › Figure6-source data 1/2.tif]

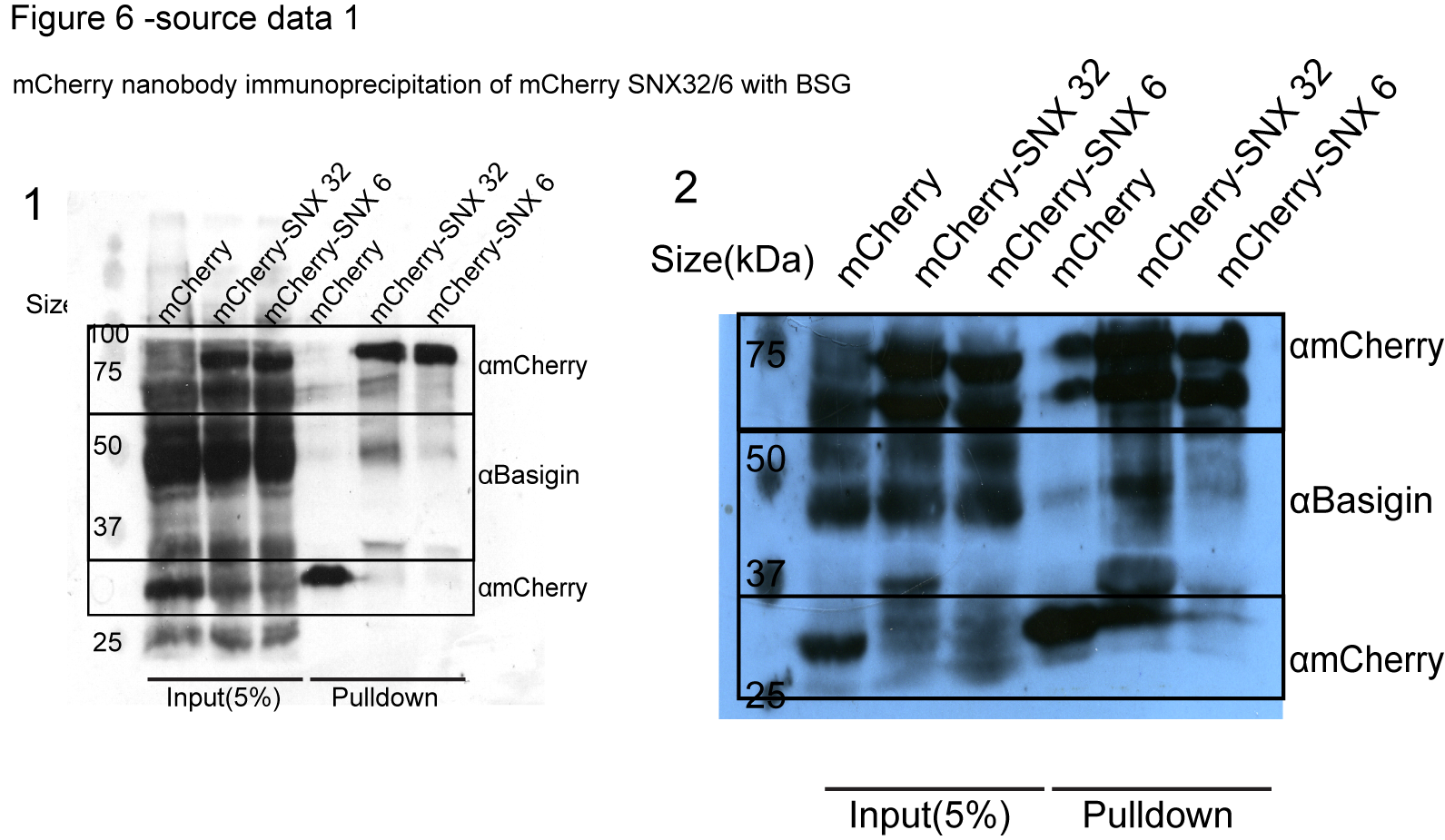

Supplement: Figure 6—source data 1. — mCherry nanobody-mediated pulldown was carried out as described in the ‘Materials and methods’ section and immunoblotted using mCherry and BSG antibody. Immunoblot source data of two biological replicates (values represent the ratio of BSG to mCherry band intensity). [file elife-84396-fig6-data1.zip › Figure6-source data 1/Figure6-source data1.tif]

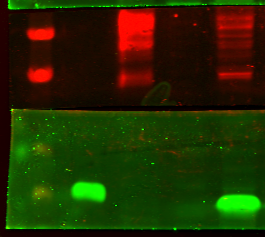

Supplement: Figure 6—source data 2. — Immunoblot source data of two biological replicates (values represent the ratio of BSG to His band intensity). [file elife-84396-fig6-data2.zip › Figure6-source data 2/1.tif]

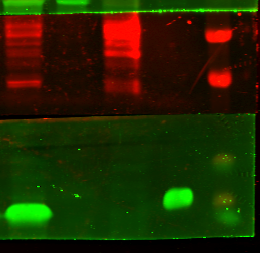

Supplement: Figure 6—source data 2. — Immunoblot source data of two biological replicates (values represent the ratio of BSG to His band intensity). [file elife-84396-fig6-data2.zip › Figure6-source data 2/2.tif]

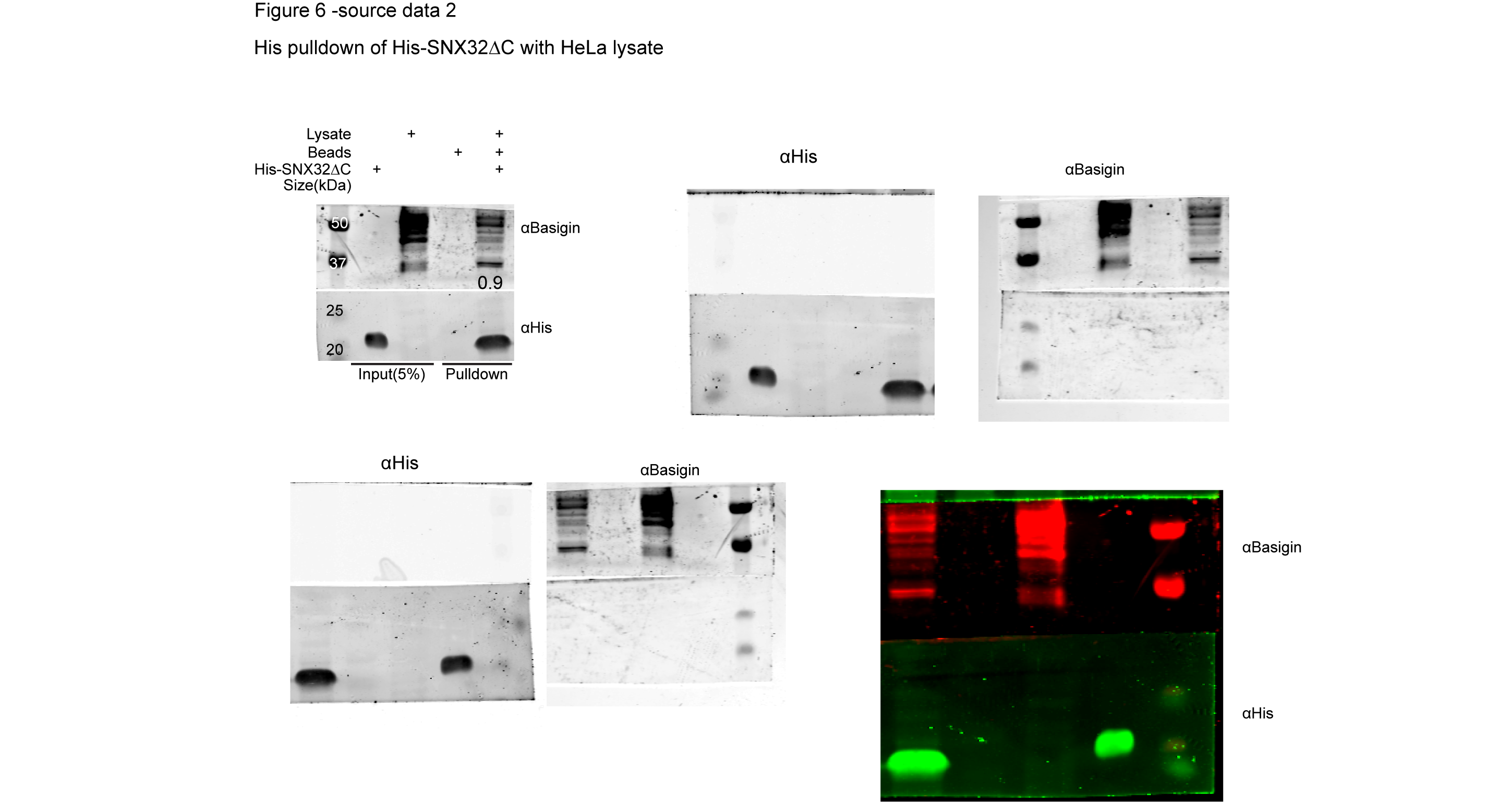

Supplement: Figure 6—source data 2. — Immunoblot source data of two biological replicates (values represent the ratio of BSG to His band intensity). [file elife-84396-fig6-data2.zip › Figure6-source data 2/Figure6-souce data2.tif]

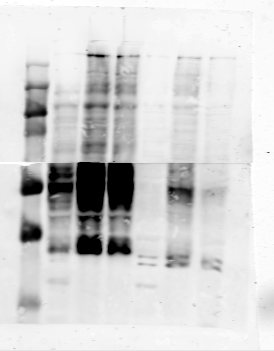

Supplement: Figure 6—source data 3. — GFP nanobody-mediated pulldown was carried out as described in the ‘Materials and methods’ section and immunoblotted using GFP and BSG antibody. Immunoblot source data of two biological replicates (values represent the ratio of BSG to GFP band intensity). [file elife-84396-fig6-data3.zip › Figure6-Source data 3/BSG_1.tif]

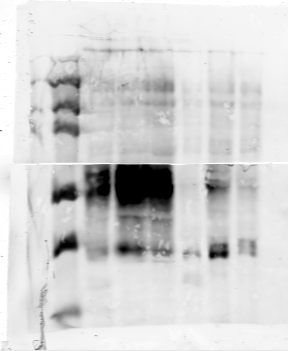

Supplement: Figure 6—source data 3. — GFP nanobody-mediated pulldown was carried out as described in the ‘Materials and methods’ section and immunoblotted using GFP and BSG antibody. Immunoblot source data of two biological replicates (values represent the ratio of BSG to GFP band intensity). [file elife-84396-fig6-data3.zip › Figure6-Source data 3/BSG_2.tif]

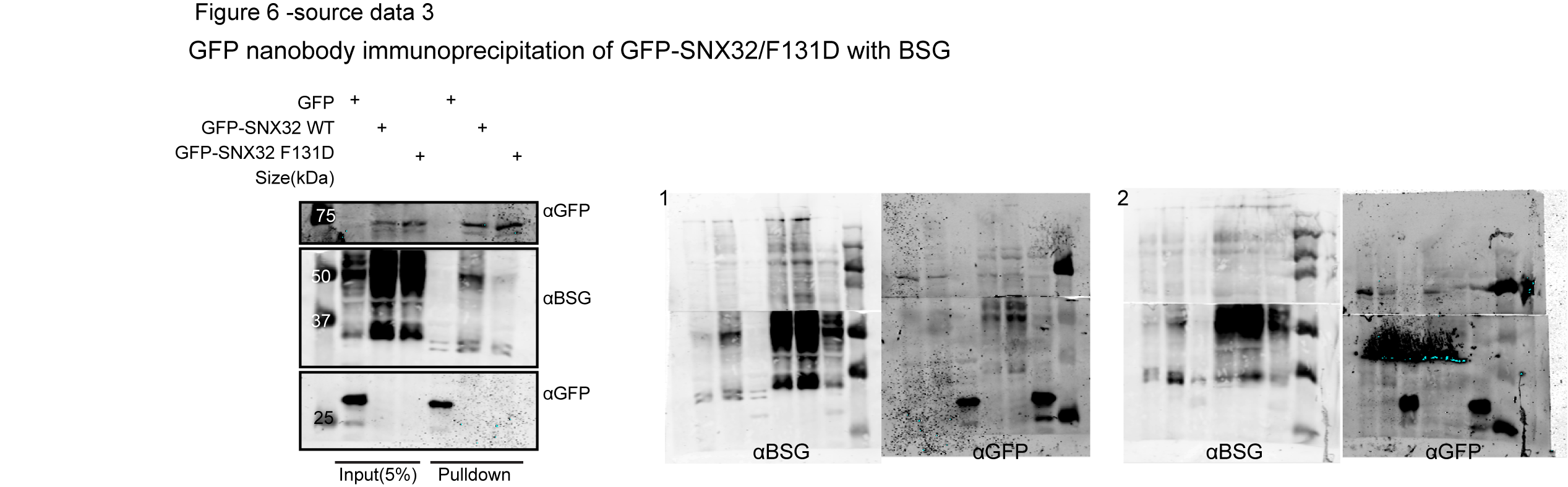

Supplement: Figure 6—source data 3. — GFP nanobody-mediated pulldown was carried out as described in the ‘Materials and methods’ section and immunoblotted using GFP and BSG antibody. Immunoblot source data of two biological replicates (values represent the ratio of BSG to GFP band intensity). [file elife-84396-fig6-data3.zip › Figure6-Source data 3/Figure6-source data 3.tif]

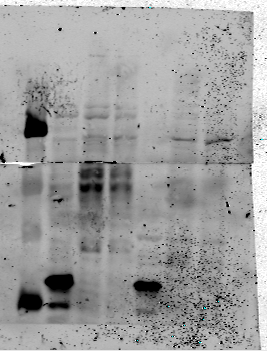

Supplement: Figure 6—source data 3. — GFP nanobody-mediated pulldown was carried out as described in the ‘Materials and methods’ section and immunoblotted using GFP and BSG antibody. Immunoblot source data of two biological replicates (values represent the ratio of BSG to GFP band intensity). [file elife-84396-fig6-data3.zip › Figure6-Source data 3/GFP_1.tif]

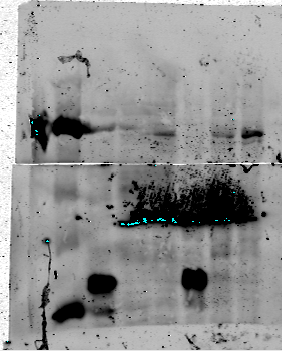

Supplement: Figure 6—source data 3. — GFP nanobody-mediated pulldown was carried out as described in the ‘Materials and methods’ section and immunoblotted using GFP and BSG antibody. Immunoblot source data of two biological replicates (values represent the ratio of BSG to GFP band intensity). [file elife-84396-fig6-data3.zip › Figure6-Source data 3/GFP_2.tif]

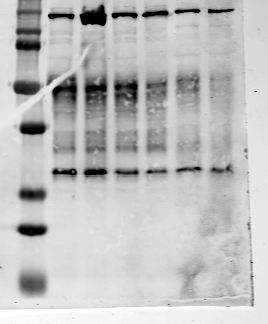

Supplement: Figure 9—figure supplement 1—source data 1. — Immunoblotting was done using BSG or vinculin antibody. Immunoblot source data of three biological replicates. [file elife-84396-fig9-figsupp1-data1.zip › Figure 9- figure supplement 1- source data 1/2.tif]

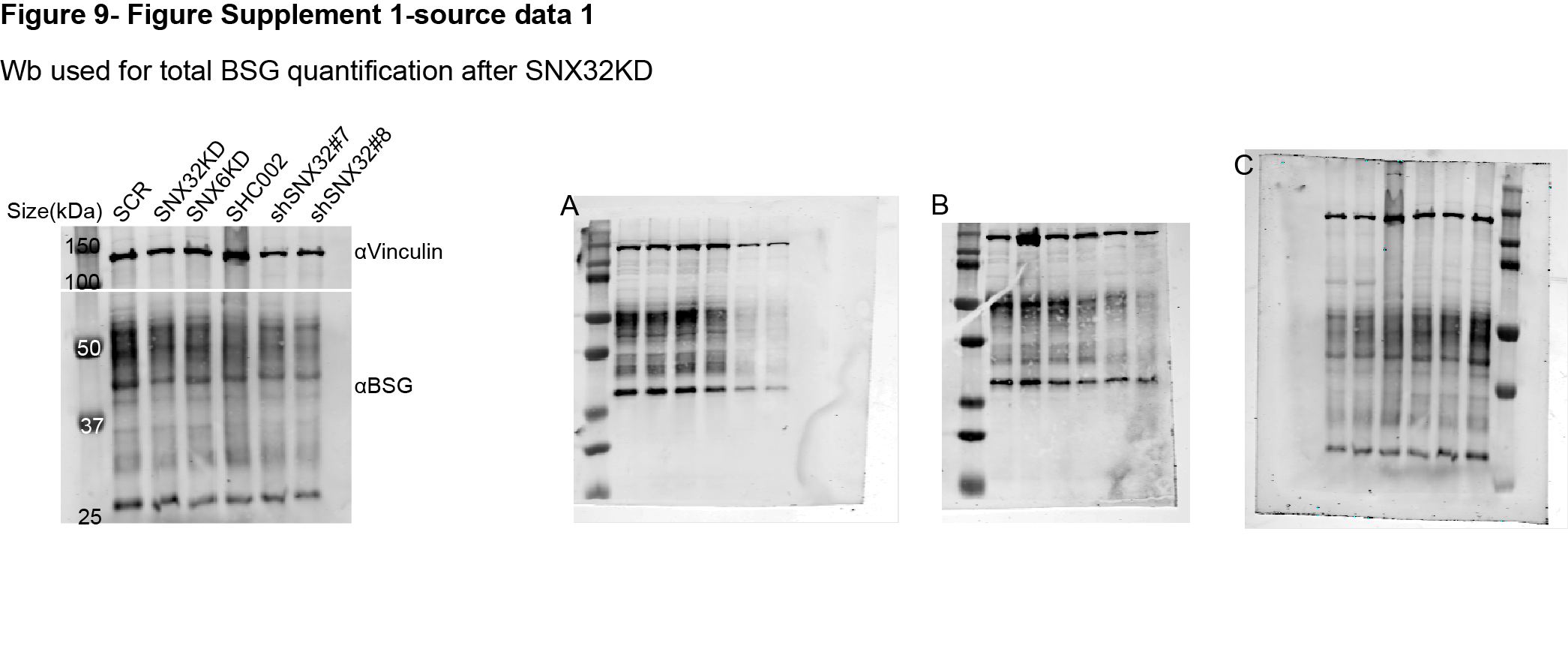

Supplement: Figure 9—figure supplement 1—source data 1. — Immunoblotting was done using BSG or vinculin antibody. Immunoblot source data of three biological replicates. [file elife-84396-fig9-figsupp1-data1.zip › Figure 9- figure supplement 1- source data 1/Figure 9- figure supplement 1- source data 1.tif]
