# Supplementary material for: Insights into cargo sorting by SNX32 and its role in neurite outgrowth
Source: eLife. 2023 May 9;12:e84396. doi: 10.7554/eLife.84396 (PMC10219652; doi:10.7554/eLife.84396)
Supplement: Figure 5—source data 5. — The elute was resolved in SDS-PAGE and immunoblotted using GFP, SNX1, and CIMPR antibody. Immunoblot source data. [file elife-84396-fig5-data5.zip › Figure5-source data 5/SNX CIMPR GFP RG.pdf]

Image ID: 0000012_01
Acquire Time: 09-Nov-2018 12:02:40

Acquisition Information

#	Image ID	Acquire Time	Channels	Resolution	Intensities	Quality	Analysis	Image Name	Comment
1	0000012_01	09-Nov-2018 12:02:40	700 800	169um	Auto Auto	lowest	Manual	0000012_01	09_11_18 IP

Image Display Values

Channel	Color	Minimum	Maximum	K
700	Red	1.93	57.2	0
800	Green	0.931	44.7	0

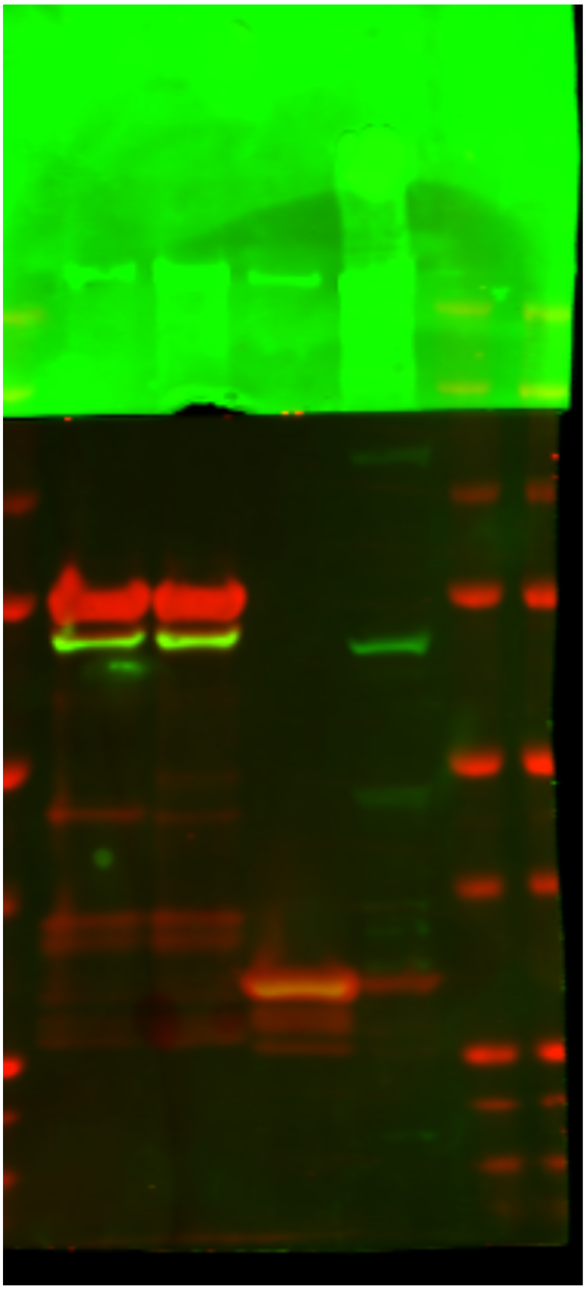

Acquisition Information (continued)

#	Image Modifications
1	
