# Supplementary material for: Insights into cargo sorting by SNX32 and its role in neurite outgrowth
Source: eLife. 2023 May 9;12:e84396. doi: 10.7554/eLife.84396 (PMC10219652; doi:10.7554/eLife.84396)

Extended data 1

Colocalization of SNX32 with TGN46 and GM130: A)Super resolution image showing co-localization of GFP-SNX32 and GM130, Scale bar 10µm, inset 1µm (magnified regions are shown as insets). B) Quantifications showing Pearson corelation coefficient co-localization of GFP-SNX32 with TGN46, GM130 in HeLa cells, data represent mean ±SEM (N=3, n≥60cells per independent experiments).C) Quantifications showing co-localization of GFP-SNX32 with TGN46,GM130 in HeLa cells calculated using automated image analysis software Motion Tracking[23,24] , data represent mean ±SEM (N=3, n≥60cells per independent experiments)

Extended data 1

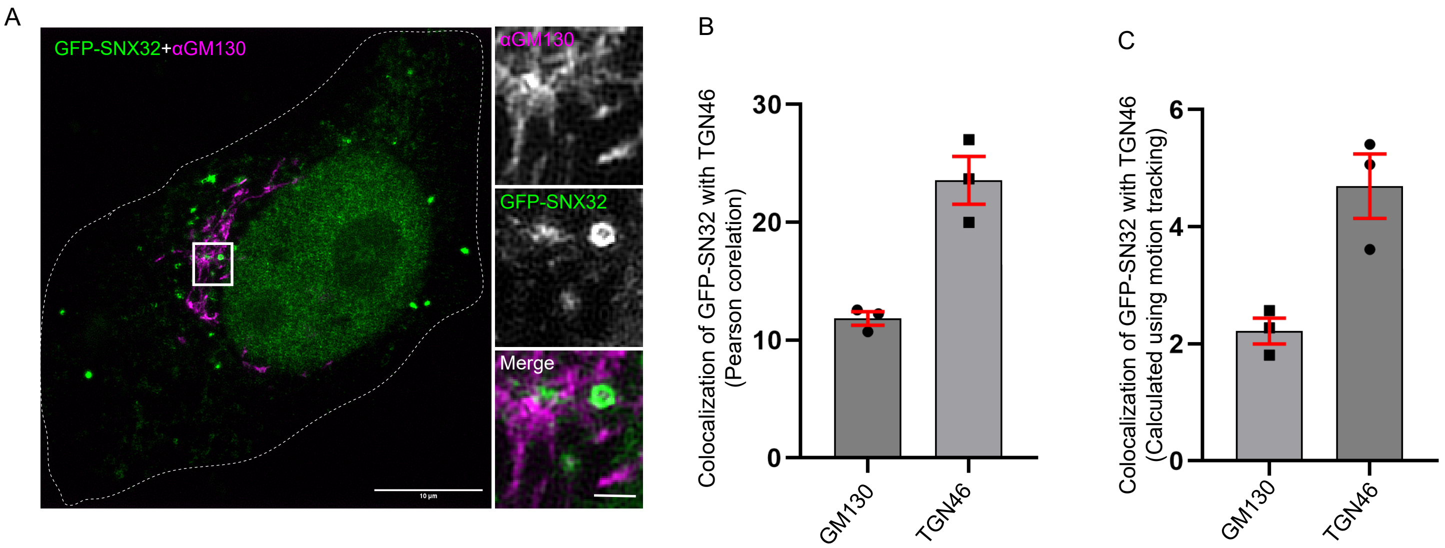

Supplement: Source data 1. [file elife-84396-data1.pdf]
