# Supplementary material for: Insights into cargo sorting by SNX32 and its role in neurite outgrowth
Source: eLife. 2023 May 9;12:e84396. doi: 10.7554/eLife.84396 (PMC10219652; doi:10.7554/eLife.84396)

Extended Data 2

Colocalization analysis : Images from each of the three independent experiments were subjected to analysis by the automated image analysis program, Motion Tracking [23, 24] (http://motiontracking.mpi-cbg.de). We identified each puncta as an object and further quantified their physical properties such as size, intensity, and area by Motion Tracking software. For better clarification, the representative image shows the EEA1 objects depicted in magenta colour and CIMPR object depicted in green colour. A) HeLa cells stained with EEA1(magenta) and CIMPR (green), identified as objects by motion tracking and B) their associated contour. C) Inset is showing the magnified boxed region with one object. D)The representative intensity distribution of this object as analyzed by motion tracking. Further, the colocalization in such a condition is calculated by analysing the total area overlap between the individual object. Objects detected in two different channels were considered colocalized if the relative overlap of respective areas was >35%. The apparent colocalization value was calculated and corrected for random colocalization.

Additional colocalization analysis was carried out using Image J image processing platform. The Pearson's correlation coefficient was determined after Costes' automatic threshold by using ImageJ-Fiji.

Extended data 2

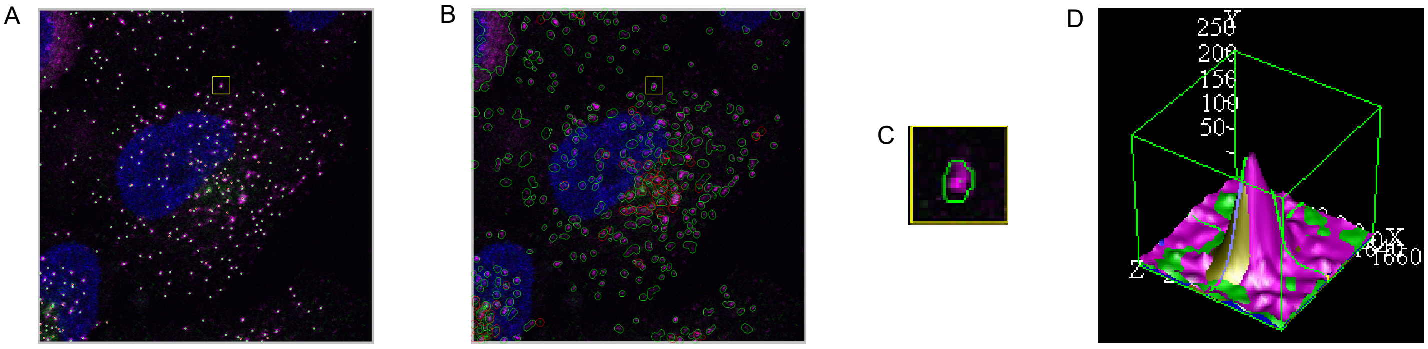

Supplement: Source data 2. [file elife-84396-data2.pdf]
